# High‐Flow Nasal Cannula in Hypercapnic Respiratory Failure: An Updated Systematic Review and Meta‐Analysis

**DOI:** 10.1111/crj.70207

**Published:** 2026-07-01

**Authors:** Yongkang Huang, Na Li, Yajuan Qian, Mengyuan Shao, Shiyuan Gao, Zengli Zhang

**Affiliations:** ^1^ Department of Respiratory and Critical Care Medicine The Second Affiliated Hospital of Soochow University Suzhou Jiangsu China; ^2^ Department of Medicine, Respiratory, Emergency and Intensive Care Medicine The Fourth Affiliated Hospital of Soochow University Suzhou Jiangsu China

**Keywords:** bi‐level positive airway pressure, high‐flow nasal cannula, hypercapnia, respiratory failure

## Abstract

**Background:**

High‐flow nasal cannula (HFNC) and bi‐level positive airway pressure (BiPAP) are both employed in the management of acute hypercapnic respiratory failure, yet their comparative benefits remain uncertain. We conducted an updated systematic review and meta‐analysis of randomized controlled trials (RCTs) to compare efficacy, safety, and tolerability of HFNC versus BiPAP in adults with hypercapnic respiratory failure.

**Methods:**

We searched PubMed, Web of Science, the Cochrane Central Register of Controlled Trials (CENTRAL), CNKI, and Wanfang Data through June 2025 for RCTs enrolling patients aged ≥ 16 years with arterial carbon dioxide partial pressure (PaCO_2_) > 45 mmHg. Primary outcomes were intubation rate and mortality. Secondary outcomes included arterial oxygen partial pressure (PaO2) or the ratio of PaO2 and inhaled oxygen fraction (FiO2; PaO_2_/FiO_2_), PaCO_2_, pH, respiratory rate, intensive care unit (ICU) length of stay, patient comfort, and device‐related complications.

**Results:**

Eleven RCTs (*n* = 1069) met inclusion criteria. Intubation (risk difference [RD] = −0.02, 95% CI −0.06 to 0.03) and mortality rates (RD = −0.02, 95% CI −0.06 to 0.02) were similar between modalities. HFNC and BiPAP showed no significant difference in PaCO_2_ reduction (mean differences [MD] = 0.74 mmHg; 95% CI, −1.21 to 2.70) or pH normalization (MD = −0.01; 95% CI, −0.01 to 0), or ICU stay (MD = −0.72 days; 95% CI, −1.90 to 0.46). However, BiPAP yielded minor statistically significant improvements in oxygenation (standardized mean differences [SMD] = 0.18, 0.03–0.33) and respiratory rate reduction (MD = 2.06; 95% CI, 1.17 to 2.94). HFNC achieved higher comfort scores and fewer skin or gastrointestinal complications.

**Conclusion:**

HFNC and BiPAP offer comparable clinical outcomes in acute hypercapnic respiratory failure. BiPAP offers modest physiological advantages, whereas HFNC provides better patient comfort and less adverse events. Large‐scale, multicenter RCTs are needed to further delineate the comparative benefits of HFNC versus BiPAP in diverse patient groups.

## Graphical abstract

HFNC and BiPAP provide comparable clinical outcomes in acute hypercapnic respiratory failure, with no significant differences in intubation or mortality. BiPAP offers modest improvements in oxygenation and respiratory rate, whereas HFNC enhances patient comfort and reduces device‐related complications. Treatment choice should balance physiological benefits against tolerability.

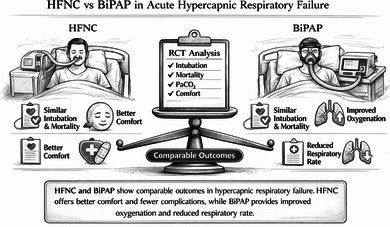

**Abbreviations**
ABG: arterial blood gases
BiPAP: bi‐level positive airway pressure
CI: confidence interval
ED: emergency department
HFNC: high‐flow nasal cannula
ICU: intensive care unit
MD: mean difference
NR: not reported
PaCO2: arterial partial pressure of carbon dioxide
PaO2: arterial partial pressure of oxygen
RD: respiratory department
RD: risk difference
RICU: respiratory ICU
SMD: standardized mean difference

## 1 Background

Hypercapnic respiratory failure arises when the respiratory system fails to adequately oxygenate arterial blood and eliminate carbon dioxide, leading to impaired gas exchange and elevated arterial carbon dioxide tension (PaCO_2_). Bi‐level positive airway pressure (BiPAP) delivers positive‐pressure ventilation via nasal, oronasal, or full‐face interfaces to enhance alveolar ventilation and reduce respiratory muscle workload. By supporting both oxygenation and CO_2_ clearance, BiPAP is recommended as first‐line therapy for acute exacerbations of chronic obstructive pulmonary disease (COPD) with hypercapnic respiratory failure, as well as for selected cases of acute cardiogenic pulmonary edema [1, 2, 3]. However, its tolerability may be limited by patient discomfort, impaired secretion clearance, restricted oral intake, and claustrophobia [4]—highlighting the importance of individualized ventilator settings and careful patient selection.

High‐flow nasal cannula (HFNC) therapy delivers heated, humidified oxygen at precisely controlled flow rates, generating a variable low‐level positive end‐expiratory pressure (PEEP) [5]. This approach improves pulmonary mechanics and enhances respiratory efficiency. Although HFNC is well established for the management of hypoxemic respiratory failure [6, 7], its role in carbon dioxide (CO_2_) elimination remains debated. Early cross‐sectional studies suggested that HFNC may facilitate CO_2_ clearance and confirmed its safety in patients with chronic CO_2_ retention [8, 9, 10]. Subsequent cohort studies demonstrated that HFNC is noninferior to noninvasive positive‐pressure ventilation (NIV) in the treatment of hypercapnic respiratory failure [11, 12]. More recently, randomized controlled trials (RCTs) have further supported these findings. However, some studies have reported that HFNC use in nonacidotic patients with hypercapnic acute exacerbations of chronic obstructive pulmonary disease (AECOPD) and elevated baseline bicarbonate levels may be associated with prolonged hospital stays—potentially due to delayed initiation of NIV [13]. Additionally, a survey of ICU physicians found that only 33% considered hypercapnic respiratory failure an appropriate indication for HFNC, and just 2% anticipated its success in this setting [14].

Our previous systematic review and meta‐analysis comparing HFNC and BiPAP in hypercapnic respiratory failure found both modalities yielded comparable clinical outcomes, although the analysis was constrained by a limited number of studies [15]. Since then, several high‐quality RCTs have been published. In the present study, we incorporate these new data to update and refine the comparative evaluation of HFNC versus BiPAP in patients with hypercapnic respiratory failure.

## 2 Methods

This updated systematic review and meta‐analysis evaluated the efficacy and safety of HFNC in hypercapnic respiratory failure based on the latest evidence. The protocol was registered at PROSPERO (www.crd.york.ac.uk/prospero; CRD: CRD420251048698) and reported in accordance with the PRISMA guideline [16].

### 2.1 Inclusion and Exclusion Criteria

The studies reviewed met the following criteria:

*Participants:* adults (age ≥ 16 years) with acute hypercapnic respiratory failure (PaCO2 > 45 mmHg).
*Type of intervention and comparator:* HFNC versus BiPAP.
*Type of studies:* parallel RCTs.
*Outcomes:* the primary efficacy outcome was the intubation rate and mortality; secondary outcomes included arterial blood‐gas parameters (PaO2 or PaO2/FiO2, PaCO2, and pH); respiratory rate; patient comfort; and device‐related complications.

Studies were excluded if (1) they used the same or overlapping data by the same authors or lacked any one of the predefined primary and secondary outcomes; or (2) non‐RCTs; or (3) studies on patients weaning from invasive mechanical ventilation.

### 2.2 Literature Search Strategy

On April 28, 2025, a trained researcher (HYK) conducted a comprehensive search of PubMed, Web of Science, Cochrane Central Register of Controlled Trials (CENTRAL), CNKI, and Wanfang Data. The search strategy combined controlled vocabulary (e.g., MeSH terms in PubMed) with free‐text keywords and synonyms related to HFNC and hypercapnic respiratory failure. Strategies were tailored to each database's indexing system. In CNKI and Wanfang, results were further filtered to include only journals listed in *A Guide to the Core Journals of China* (PKU filter). To minimize retrieval bias, reference lists of all included studies were manually screened for additional eligible trials. Full search strategies are provided in Appendix S1.

### 2.3 Study Selection

Two independent reviewers (HYK and LN) screened studies for eligibility. Duplicate records were identified and removed by cross‐checking authorship, titles, and publication dates. Titles and abstracts of remaining citations were screened against the inclusion criteria. Full‐text articles of potentially eligible studies were retrieved and assessed in detail. Disagreements were resolved through consensus, and unresolved cases were adjudicated by a third reviewer (SMY).

### 2.4 Quality Assessment

Risk of bias for each included RCT was independently assessed by two reviewers (LN and QYJ) using the Cochrane Risk of Bias Tool [17]. Domains evaluated included: random sequence generation, allocation concealment, blinding of participants and personnel, blinding of outcome assessment, incomplete outcome data, selective reporting, and other potential biases. Discrepancies were resolved through discussion or adjudicated by a third reviewer (SMY).

### 2.5 Data Extraction

Two reviewers (GSY and SMY) independently extracted data using a standardized form. Extracted information included as follows: (1) Study characteristics: first author, publication year, country, trial registration number, and journal. (2) Design details: sample size, trial setting (e.g., ICU and ED), cause of hypercapnic respiratory failure, major inclusion criteria, HFNC flow rate setting, and follow‐up duration. (3) Participant demographics: age, sex, baseline arterial blood‐gas values (PaO_2_, PaCO_2_, and pH), and other key clinical metrics. (4) Outcomes: primary endpoints (intubation rate and mortality) and secondary endpoints (PaO_2_ or PaO_2_/FiO_2_, PaCO_2_, pH, respiratory rate, comfort scores, device‐related complications, and ICU/hospital length of stay).

When both intention‐to‐treat and per‐protocol analyses were presented, we prioritized intention‐to‐treat results. Discrepancies were resolved by discussion; unresolved disagreements were adjudicated by a third reviewer. If data such as mean and SDs values were missing or unclear, we contacted corresponding authors via email; when no reply was received, we estimated values from available information using validated formulas [18].

### 2.6 Statistical Analysis

All statistical analyses were performed by an independent author using Review Manager (RevMan) version 5.4 (Cochrane Collaboration) and R version 4.1.1 (R Foundation). Continuous outcomes were pooled as mean differences (MDs) with 95% confidence intervals (CIs) when units were consistent; otherwise, standardized mean differences (SMDs) were used. Dichotomous outcomes were pooled as risk differences (RDs) with 95% CIs.

Heterogeneity was assessed using Cochran's *χ* ^2^ test (significance threshold: *p* < 0.10) and the *I* ^2^ statistic (*I* ^2^ > 50% indicating substantial heterogeneity). A fixed‐effect model was applied in the absence of heterogeneity; otherwise, a random‐effects model was used. For primary efficacy outcomes, Galbraith radial plots were generated, and meta‐regression was conducted when data permitted to explore sources of heterogeneity. Pooled estimates were visualized using forest plots. Sensitivity analyses were performed to assess the robustness of findings. Publication bias was evaluated using funnel plots, Egger's test, and Duval and Tweedie's trim‐and‐fill method, where applicable.

## 3 Result

### 3.1 Study Selection and Characteristics

**FIGURE 1:**
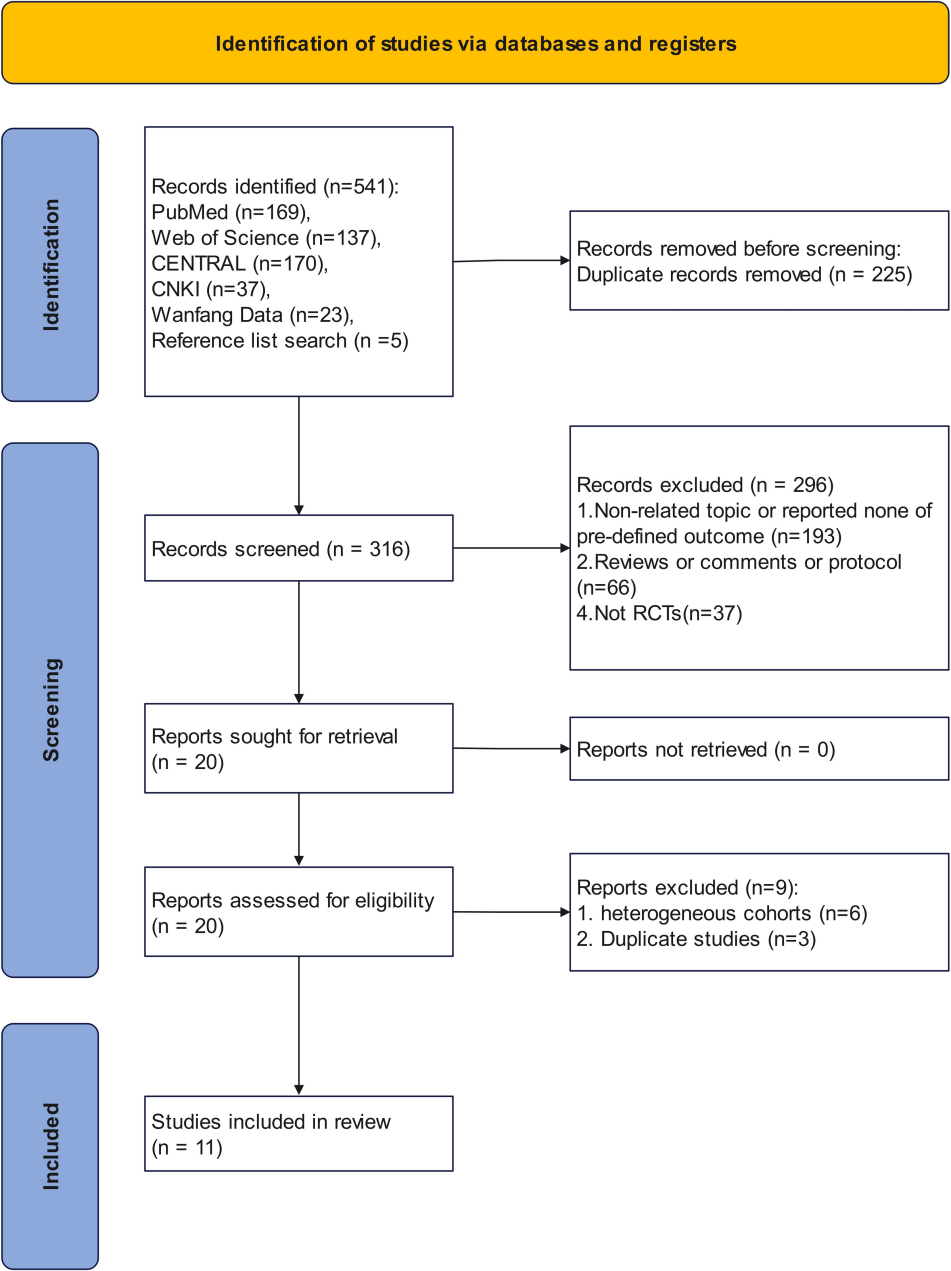
Study flow.

A total of 541 records were identified through the literature search. After removing 225 duplicates based on title and author information, 316 unique records remained for title and abstract screening. Of these, 296 were excluded for not meeting the predefined inclusion criteria, and full texts were retrieved for the remaining 20 articles. After removing three duplicated studies and six studies with heterogeneous cohorts, 11 parallel‐group RCTs [19, 20, 21, 22, 23, 24, 25, 26, 27, 28, 29], comprising 1069 participants, were ultimately included in the quantitative synthesis. The detailed identification and screening process is illustrated in Figure 1.

Among the included studies, three were multicenter trials and eight were single‐center studies conducted in emergency departments, intensive care units (ICUs), or respiratory ICUs (RICUs). Four trials were prospectively registered. Six studies (54.5%) were conducted in China—four published in Chinese and two in English—whereas the remaining were conducted in other countries. One study did not specify the etiology of hypercapnic respiratory failure, although most cohorts consisted of patients with AECOPD. HFNC flow rates ranged from 25 to 60 L/min and were titrated based on patient comfort. Detailed participant demographics and study characteristics are presented in Tables 1 and 2.

**TABLE 1:** Characteristics of the studies.

Authors	Case souce	Number	Country	Type	Major inclusive criteria	Outcome	Follow‐up (days)	Trial registration number	HFNC setting
Elhofy et al. 2025	RICU	28	Egypt	Single‐center RCT	≥ 18 years, acute hypercapnic respiratory failure	Primary: clinical improvement, PCO_2_, and pH trends secondary: deterioration, dyspnea (Borg score), distress symptoms, and unresolved distress	28	NR	25–40 L/min
Haciosman et al. 2025	ED	137	Republic of Türkiye	Single‐center RCT	≥ 18 years, COPD, ED presentation with bronchodilator‐resistant exacerbation	Primary: blood gas changes secondary: invasive support, ICU admission, and mortality	28	NCT06495086	30 L/min OR 50 L/min
Tan et al. 2024	ICUs	225	China	Single‐center RCT	AECOPD, moderate hypercapnic ARF (pH 7.25–7.35, PaCO_2_ ≥ 50 mmHg)	Primary: treatment failure secondary: invasive ventilation, switch, vitals, ABG (1/12/48 h), 28‐day mortality, and ICU/hospital stay	28	ChiCTR1800014553	40 L/min
Pantazopoulos et al. 2024	ED and RD	105	Greece	Multicenter RCT	COPD with mild‐to‐moderate acute/acute‐on‐chronic hypercapnic RF, RR > 23 after bronchodilators and oxygen	Primary: treatment failure (intubation/switch) secondary: respiratory changes, comfort, and pulmonary/extrapulmonary issues	NR	NCT03466385	50–60 L/min
Tang et al. 2023	RD	84	China	Single‐center RCT	COPD patients with type II respiratory failure	Symptom progression and ABG changes	30	NR	35–60 L/min
Bian 2022	NR	100	China	Single‐center RCT	COPD patients with type II respiratory failure	Symptom progression, ABG changes, comfort, and complications	NR	NR	40–60 L/min
Papachatzakis et al. 2020	ED	40	Greece	Single‐center RCT	Patients with acute type II respiratory failure	Intubation, mortality, hospital/therapy duration, vital signs, ABG, and comfort	NR	NR	45–50 L/min
Cortegiani et al. 2020	ED, RD, ICU	79	Italy	Multicenter RCT	> 18 years, COPD exacerbation (GOLD), and mild‐to‐moderate hypercapnic RF (pH 7.25–7.35, PaCO_2_ ≥ 55 mmHg)	Primary: PaCO_2_ difference at 2 h secondary: HFNT noninferiority (6‐h PaCO_2_), treatment switch, dyspnea/discomfort, RR, ABG changes, IMV/NIV duration, and hospital stay/mortality	NR	NCT03370666	60 L/min
Liu et al. 2019	RD	60	China	Single‐center RCT	COPD patients with type II respiratory failure	ABG and vital sign changes	NR	NR	20‐40 L/min
Wang et al. 2019	RICU	43	China	Single‐center RCT	AECOPD with hypercapnic respiratory failure	Treatment failure (switch/intubation), tracheal intubation, complications, and 28‐day survival	28	NR	NR
Cong et al. 2019	ICU	168	China	Single‐center RCT	AECOPD patients	Primary: ABG secondary: support duration, hospital stay, complications, comfort, and nursing satisfaction	NR	NR	30‐35 L/min

**TABLE 2:** Characteristics of the participants.

Authors	Age (years)		Gender (male/totle)		APACHE II score		Respiratory rates (times/min)		pH		PaO2(mmHg) or PaO2/FiO2* (mmHg)		PaCO2 (mmHg)		Respiratory support duration (days or hours*)	
	HFNC	BiPAP	HFNC	BiPAP	HFNC	BiPAP	HFNC	BiPAP	HFNC	BiPAP	HFNC	BiPAP	HFNC	BiPAP	HFNC	BiPAP
Elhofy et al. 2025	56.79 ± 11.48	62.33 ± 8.3	11/24	11/24	21(20.0–22.0)	21.5(21–25)	30 ± 3	31 ± 3	7.35 ± 0.07	7.31 ± 0.07	77.7 ± 7.8	76.3 ± 7.5	59.9 ± 8.5	66.4 ± 12.9	48 (48–72)*	48 (5–72)*
Haciosman et al. 2025, 30 L/min	67.6 ± 11.4	68.8 ± 10.5	30/46	31/47	—	—	28.78 ± 2.72	27.94 ± 3.28	7.3 ± 0.04	7.3 ± 0.04	—	—	62.29 ± 9.87	64.69 ± 10.81	—	—
Haciosman et al. 2025, 50 L/min	67.7 ± 9.7	68.8 ± 10.5	29/44	31/47	—	—	29.25 ± 2.77	27.94 ± 3.28	7.32 ± 0.03	7.3 ± 0.04	—	—	61.51 ± 9.03	64.69 ± 10.81	—	—
Tan et al. 2024	73(65–78)	69(63–76)	71/113	62/112	14(11–17)	12(10–16)	28(25–30)	29(26–32)	7.31(7.29–7.33)	7.30(7.28–7.32)	175(167–199)	184(167–202)	63(59–68)	61(58–65)	85.9 ± 30.5*	78.7 ± 33.8*
Pantazopoulos et al. 2024	72.37 ± 9.18	73.26 ± 9.5	34/51	41/54	—	—	30 (6)	32.5(10)	7.3 (0.03)	7.29 (0.05)	237.78(58.27)	238.1(43.19)	60.8(10)	65(13)	48(48)*	60(48)*
Tang et al. 2023	64.68 ± 7.27	65.05 ± 6.98	23/42	19/42	—	—	30.4 ± 3.67	29.95 ± 3.41	—	—	55.33 ± 6.43	54.76 ± 5.96	70.17 ± 8.37	71.36 ± 7.19	—	—
Bian 2022	—	—	—	—	—	—	—	—	7.31 ± 0.08	7.32 ± 0.09	55.29 ± 4.86	54.87 ± 5.02	56.79 ± 6.12	57.18 ± 6.34	8.12 ± 1.62	7.86 ± 1.34
Papachatzakis et al. 2020	76.0 ± 13.4	78.1 ± 8.1	10/20	10/20	21.6 ± 8.9	19.3 ± 6.1	21.3 ± 8.7	26.6 ± 5.3	7.4 ± 0.1	7.4 ± 0.1	65.2 ± 12.9	71.6 ± 19.8	60.4 ± 9.9	62.1 ± 10.3	2 ± 1	2 ± 9
Cortegiani et al. 2020	74 ± 13	77 ± 12	21/40	19/39	—	—	27 ± 7	28 ± 7	7.3 ± 0.03	7.29 ± 0.03	64.3 ± 17.6	73.3 ± 27.9	73.7 ± 12.8	72 ± 13	—	—
Doshi et al. 2020	65(56–73)	59(59–70)	15/34	16/31	31(28–34)	29(26–34)	32(28–36)	28(24–32)	7.33(7.24–7.40)	7.32(7.26–7.39)	98.5(84–168)	98(62–125)				
Liu et al.2019	66 ± 9.36	67 ± 9.78	16/30	15/30	—	—	—	—	—	—	48.58 ± 4.36	47.35 ± 4.25	65.43 ± 5.28	65.37 ± 5.43	—	—
Wang et al. 2019	71.26 ± 7.39	72.85 ± 6.65	13/23	12/20	18.35 ± 2.19	18.9 ± 2.59	30.91 ± 2.13	30.35 ± 2.68	7.23 ± 0.19	7.24 ± 0.02	57.17 ± 5.68	59.55 ± 6.48	67.13 ± 4.25	66.05 ± 3.03	7.96 ± 1.72	6.8 ± 1.26
Cong et al. 2019	66.91 ± 7.38	67.88 ± 8.38	48/84	50/48	—	—	—	—	7.25 ± 0.08	7.27 ± 0.09	53.10 ± 16.22	54.08 ± 15.33	72.11 ± 16.31	72.91 ± 16.41	10.02 ± 5.11	9.55 ± 4.78

### 3.2 Risk of Bias Within Studies

**FIGURE 2:**
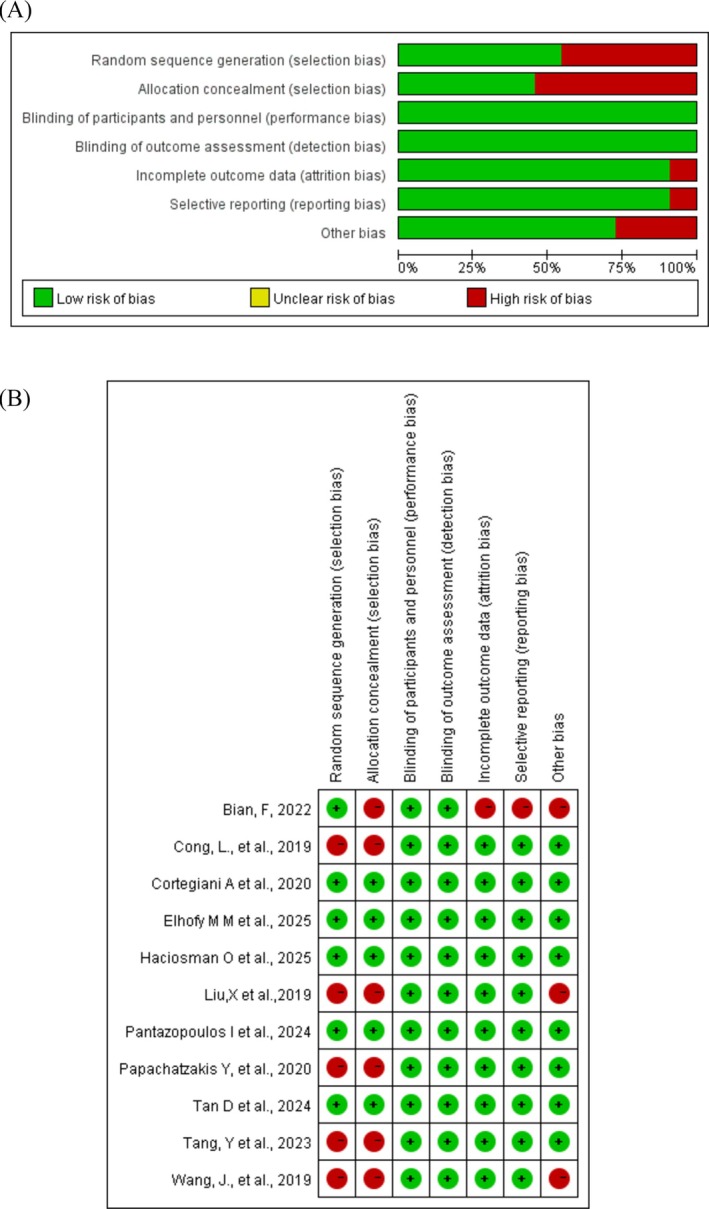
Risk of bias of the included studies (A) Risk of bias graph: review authors judgments about each risk of bias item presented as percentages across all included studies; (B) risk of bias summary: review authors judgments about each risk of bias item for each included study.

Assessment using the Cochrane Risk of Bias tool revealed that five trials did not report methods for random sequence generation or allocation concealment, indicating potential selection bias. Due to the inherent differences between HFNC and BiPAP interfaces, none of the studies blinded participants or personnel. The trial by Bian et al. lacked baseline participant characteristics and listed only a single author, raising concerns about reporting and other biases. Similarly, Wang et al. did not specify the cause of admission, and Liu et al. listed only three authors, suggesting possible undisclosed biases. No evidence of attrition bias was found across the included trials. A summary of the quality assessment is provided in Figure 2.

### 3.3 Clinical Effectiveness

#### 3.3.1 Intubation Rate and Mortality

**FIGURE 3:**
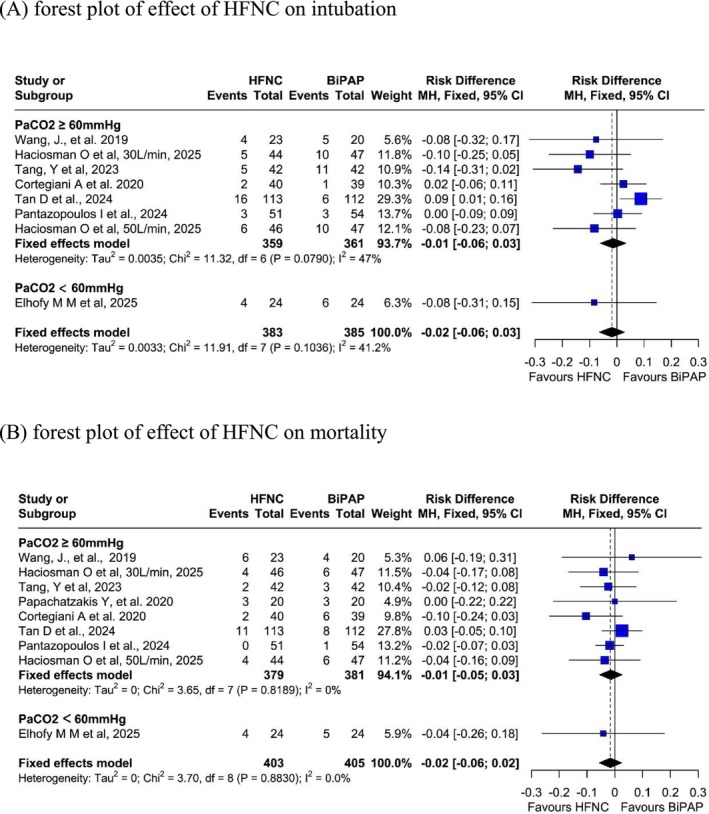
Effect on intubation rate and mortality (A) forest plot of effect of HFNC on intubation and (B) forest plot of effect of HFNC on mortality.

Seven RCTs (*n* = 721) reported intubation rates. Pooled analysis showed HFNC was comparable to BiPAP in preventing intubation (RD = −0.02; 95% CI, −0.06 to 0.03). Eight RCTs (*n* = 761) reported mortality outcomes, with no significant difference between HFNC and BiPAP (RD = 0.02; 95% CI, −0.06 to 0.02). Forest plots are shown in Figure 3.

#### 3.3.2 Blood Gas Analysis and Respiratory Rate

Among studies reporting blood‐gas parameters (PaO_2_, PaCO_2_, pH) and respiratory rate approximately 48 h after intervention initiation, data were pooled and analysed.

**FIGURE 4:**
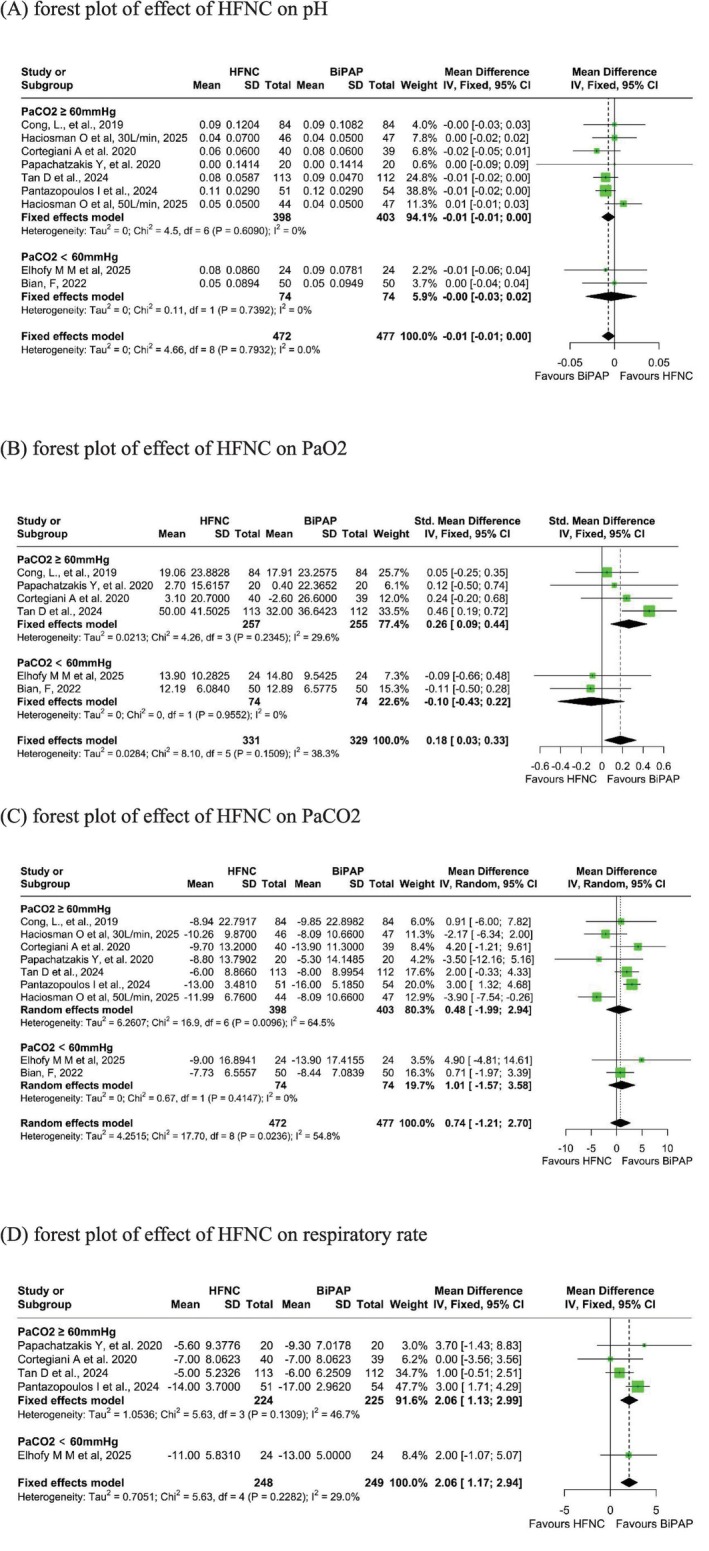
Effect on blood gas analysis and respiratory rate (A) forest plot of effect of HFNC on pH, (B) forest plot of effect of HFNC on PaO2, (C) forest plot of effect of HFNC on PaCO2, and (D) forest plot of effect of HFNC on respiratory rate.

Across 8 RCTs (*n* = 902), there was no significant difference between HFNC and BiPAP in PaCO_2_ reduction (MD = 0.74 mmHg; 95% CI –1.21 to 2.70) and pH normalization (MD = −0.01; 95% CI –0.01 to 0; 8 RCTs, *n* = 902). In contrast, BiPAP conferred small but statistically significant advantages in oxygenation (SMD = 0.18; 95% CI 0.03 to 0.33; 6 RCTs, *n* = 660), and reduction of respiratory rate (MD = 2.06 breaths/min; 95% CI 1.17 to 2.94; 5 RCTs, *n* = 497). Forest plots summarizing these comparisons are presented in Figure 4.

#### 3.3.3 ICU Stay

**FIGURE 5:**
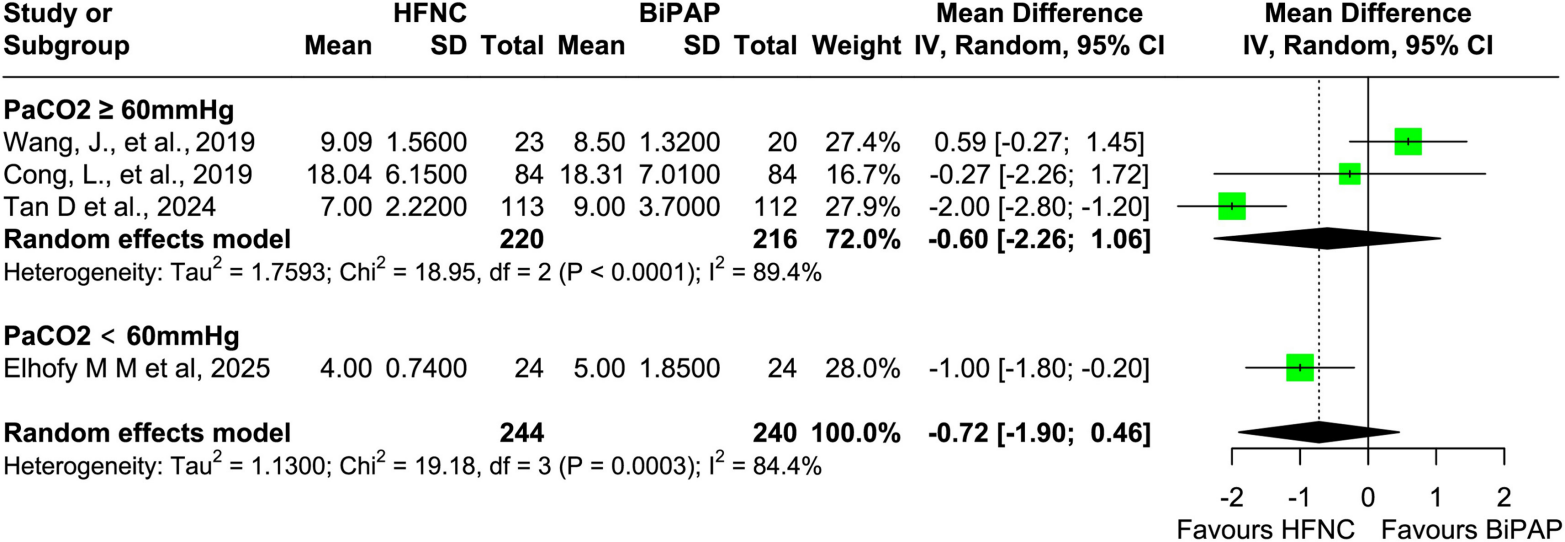
Effect on ICU stay.

Four RCTs reporting ICU length of stay were pooled. Overall, HFNC did not significantly alter ICU stay compared with BiPAP (MD = −0.72 days; 95% CI, −1.90 to 0.46). Forest plots for ICU stay are presented in Figure 5.

#### 3.3.4 Patient Comfort and Complications of Therapy

Nine RCTs assessed patient comfort and device‐related complications, including nasal/facial skin breakdown, gastrointestinal distension, and tolerability comfort scores. Every trial reported statistically significant advantages for HFNC over BiPAP in the aspects mentioned above. Specifically, HFNC was associated with a lower incidence of gastrointestinal distension (range 2.0%–26.2% vs. 4.0%–45.0%) and nasal/facial skin breakdown (0%–8.7% vs. 8.0%–40.0%), as well as higher mean tolerability comfort scores and better tolerance. A comprehensive summary of these comfort and complication outcomes is provided in Table 3.

**TABLE 3:** Patients' comfort and complications.

Authors	Comfort scores	Gastric and intestinal flatulence	Nasal facial skin breakdown	Failure to tolerate modality
Elhofy et al. 2025	—	—	—	0/24 vs. 3/24
Haciosman et al. 2025	1.63 ± 1.02 vs. 2.09 ± 1.25, *p* < 0.05	—	—	—
Haciosman et al. 2025	3.84 ± 1.12 vs. 2.91 ± 1.25, *p* < 0.05	—	—	—
Pantazopoulos et al. 2024	6 ± 0.74 vs. 9.05 ± 1.5, *p* < 0.05	—	—	
Tang et al. 2023	—	26.2% vs. 9.5%, *p* < 0.05	14.3% vs. 0, *p* < 0.05	—
Bian 2022	89.62 ± 3.61 vs. 72.58 ± 4.25	1/50 vs. 2/50	4/50 vs. 0	—
Papachatzakis et al. 2020	—	—	—	0 vs. 3/20
Cortegiani et al. 2020	0[0–2] vs. 2[1–4]	—	—	—
Wang et al. 2019	—	13.0% vs. 45.0%, *p* = 0.039	8.7% vs. 40%, *p* = 0.028	—
Cong et al. 2019	2.52 ± 0.75 vs. 2.83 ± 0.51, *p* = 0.008	—	—	—

### 3.4 Sensitivity Analysis

Sensitivity analyses were conducted to assess the robustness of our efficacy findings, including subgroup analyses, changing statistical models, exclusion of low‐quality studies, and leave‐one‐out analysis.

#### 3.4.1 Subgroup Analyses

Subgroup analyses were stratified by PaCO_2_ levels. Studies in which the mean PaCO_2_ in the HFNC group was ≥ 60 mmHg were categorized as high PaCO_2_; all others were classified as low PaCO_2_. The subgroup results closely aligned with the overall analysis. Notably, BiPAP demonstrated a greater improvement in oxygenation within the high PaCO_2_ subgroup (SMD = 0.26; 95% CI, 0.09–0.44; 4 RCTs, *n* = 512), whereas outcomes in the low PaCO_2_ subgroup were comparable between interventions (SMD = −0.10; 95% CI, −0.43–0.22; 2 RCTs, *n* = 148). Reductions in respiratory rate were also more pronounced in the high PaCO_2_ subgroup (MD = 2.06; 95% CI, 1.13–2.99) compared to the low PaCO_2_ subgroup (MD = 2.00; 95% CI, −1.07–5.07).

#### 3.4.2 Alternative Statistical Models

All endpoints were reanalyzed using alternative statistical models. As shown in Table 4, most effect estimates remained consistent. The primary outcomes—intubation rate and mortality—preserved both their magnitude and statistical significance. However, pooled PaCO_2_ results differed by model: under the fixed‐effect model, BiPAP significantly reduced PaCO_2_ (MD = 1.44; 95% CI, 0.38–2.50), whereas the random‐effects model did not yield a significant difference (MD = 0.74; 95% CI, −1.21–2.70). The direction of effect for PaO_2_ and ICU stay remained unchanged, although statistical significance was reversed under the alternative model.

**TABLE 4:** Sensitivity analysis.

	Original (fixed or random)	Alternative model (fixed or random)	≥ 2 high risk studies omitted
Intubation	−0.02 (−0.06, 0.03)	−0.02 (−0.08, 0.04)	0 (−0.05, 0.05)
Mortality	−0.02 (−0.06, 0.03)	−0.02 (−0.05, 0.02)	−0.02 (−0.06, 0.02)
pH	−0.01 (−0.01, 0)	−0.01 (−0.01, 0)	−0.01 (−0.01, 0)
PaO2	0.18 (0.03, 0.33)	0.15 (−0.07, 0.36)	0.33 (0.12, 0.54)
PaCO2	0.74 (−1.21, 2.7)	1.44 (0.38, 2.50)	0.96 (−1.77, 3.68)
Respiratory rate	2.06 (1.17, 2.94)	1.93 (0.65, 3.21)	2.0 (1.10, 2.91)
Stay of ICU	−0.72 (−1.90, 0.46)	−0.84 (−1.30, −0.38)	−1.50 (−1.48, −0.52)

#### 3.4.3 Exclusion Of Low‐Quality Studies And Leave‐One‐Out Analysis

To assess the influence of study quality, we excluded trials with two or more domains rated as high risk of bias and repeated the meta‐analysis. Although point estimates shifted slightly, overall trends and significance levels remained stable—except for ICU stay, where HFNC maintained the same direction of effect but reached statistical significance after excluding high‐risk studies (Table 4).

**FIGURE 6:**
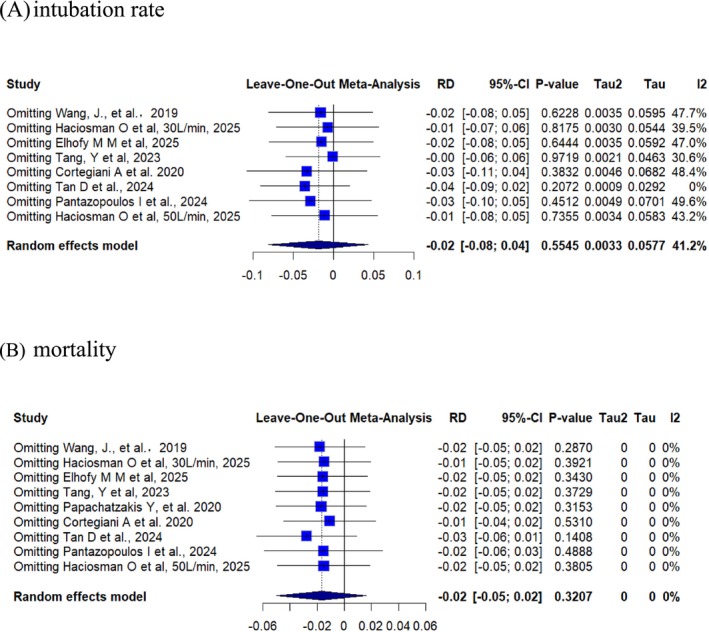
Sensitivity analysis The included studies were resynthesized by omitting one at a time on intubation (A) and mortality (B). Intubation rate mortality.

Leave‐one‐out analyses, in which each study was sequentially omitted, were performed across the available meta‐analyses. These confirmed that no single trial disproportionately affected the pooled effect sizes for most outcomes. This supports the robustness of our findings. However, for PaCO_2_ and pH, statistical significance emerged only after removing the study by Haciosman O et al. (50‐L/min subgroup) (Figure 6; Figures S3–S7).

### 3.5 Heterogeneity Analysis

Heterogeneity was acceptable for most outcomes but substantial for PaCO_2_, respiratory rate, and ICU length of stay. Meta‐regression was performed to explore sources of PaCO_2_ heterogeneity using HFNC flow settings (unreported, low, hybrid, and high) and PaCO_2_ level (high vs. low); neither covariate significantly explained the variance (*p* > 0.05). Radial plot analysis (Figure S1) identified the subgroup trial by Haciosman et al., which used a 50‐L/min flow rate, as a potential source of heterogeneity. For ICU length of stay, the limited number of studies precluded meta‐regression; radial plots suggested trials by Tan et al. and Wang et al. as contributors.

### 3.6 Publication Bias

**FIGURE 7:**
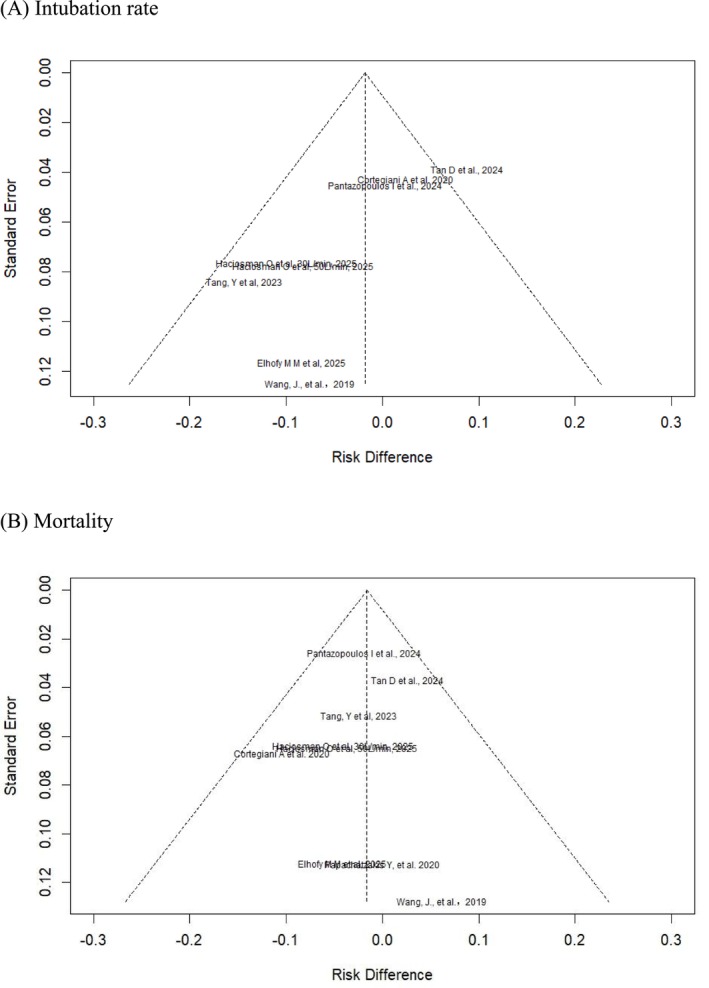
Funnel plots of HFNC on the intubation rate and mortality. (A) intubation rate and (B) mortality.

Given the limited number of included studies (*n* < 10), Egger's regression test was deemed inappropriate for assessing publication bias. Instead, funnel plots (Figure 7) were visually examined and appeared slightly asymmetrical for both intubation and mortality outcomes. To further evaluate potential bias, Duval and Tweedie's trim‐and‐fill method was applied. The adjusted results remained consistent with the original pooled estimates, suggesting minimal impact of publication bias. Specifically, for intubation, the risk difference (RD) was 0.033 (95% CI, −0.032 to 0.099) after imputing four hypothetical studies. For mortality, the adjusted RD was −0.011 (95% CI, −0.043 to 0.021) with one study added.

## 4 Discussion

In this updated synthesis, we systematically reviewed RCTs published since our previous analysis. To enhance methodological rigor and minimize bias, this meta‐analysis excluded cohort studies that were included in the original review [11, 12], given the well‐documented influence of study design on pooled outcomes. Additionally, studies evaluating noninvasive ventilation or HFNC in the postextubation setting [25, 30, 31] were also excluded in this study due to significant population heterogeneity. Interventions targeting acute hypercapnic respiratory failure differ substantially in purpose and patient profile from those aimed at preventing postextubation respiratory failure, warranting separate consideration.

In contrast to previously published meta‐analyses, our study employed more stringent inclusion criteria to enhance internal validity and reduce clinical heterogeneity. Specifically, we required a baseline PaCO_2_ > 45 mmHg to ensure a consistently hypercapnic population. This threshold led to the exclusion of trials such as those by Francio et al. [32] (HFNC group PaCO_2_ IQR: 46–73 mmHg) and Doshi et al. [33]. (PaCO_2_ range: 26–112 mmHg), which included normocapnic patients. Notably, these studies were incorporated into prior meta‐analyses [34, 35], potentially diluting effect estimates and limiting the applicability of their findings to hypercapnic cohorts. By narrowing our focus, our analysis offers more clinically relevant insights into the efficacy of noninvasive respiratory support in patients with confirmed hypercapnia.

Consistent with our earlier findings, pooled data confirmed that HFNC and BiPAP are associated with comparable rates of tracheal intubation and mortality. However, in this updated meta‐analysis, BiPAP showed superior efficacy in improving oxygenation and reducing respiratory distress. These benefits were most evident in patients with higher baseline PaCO_2_ levels (≥ 60 mmHg) and did not reach statistical significance in those with PaCO_2_ < 60 mmHg. This disparity may be attributable to the relatively conservative fraction of inspired oxygen typically administered during HFNC in patients with severe hypercapnia, whereas more aggressive ventilatory settings, including higher pressure support levels, are often applied during noninvasive ventilation in this population.

Our subgroup analyses highlight the influence of baseline PaCO_2_ on clinical outcomes, providing insight into patient stratification and guiding individualized therapy and future research directions.

The primary endpoints—intubation rate and mortality—showed minimal between‐study heterogeneity and remained robust across sensitivity and influence analyses. Secondary outcomes such as PaCO_2_, PaO_2_, and ICU length of stay demonstrated less stability. Although the direction of effect remained consistent in alternative models or after exclusion of low‐quality studies, statistical significance occasionally reversed. This inconsistency might be attributable to substantial between‐study heterogeneity and differences in statistical modeling. Fixed‐effect models assume a common underlying treatment effect across studies and may underestimate uncertainty when heterogeneity is present, resulting in artificially narrow confidence intervals. In contrast, random‐effects models account for between‐study variability, yielding wider confidence intervals and more conservative estimates. Given the significant heterogeneity observed for several secondary outcomes, the random‐effects model is likely more appropriate. Therefore, findings that achieved statistical significance only under the fixed‐effect model should be interpreted cautiously and should not be considered definitive evidence of treatment benefit.

In exploring the possible source of PaCO_2_ heterogeneity, radial plot analysis identified the subgroup trial by Haciosman et al. as a potential source of heterogeneity. This could be caused by an actual higher HFNC flow rate setting (50 L/min) and a higher carbon dioxide clearance rate compared to about 40 L/min in other studies. HFNC's proposed mechanisms—pharyngeal dead‐space washout and correction of hypoxemia—optimize alveolar gas exchange and may account for its effect on PaCO_2_ reduction. Flow rate is a key determinant of HFNC performance. Higher flow rates enhance oxygenation and facilitate dead‐space clearance but may increase patient discomfort and air leakage, particularly in patients who breathe through the mouth. When the mouth remains closed, elevated flow can also increase PEEP, improving respiratory mechanics [36, 37, 38, 39]. Current evidence supports initiating HFNC at flow rates above 30 L/min for patients with COPD and hypercapnia [40, 41].

Nevertheless, several limitations of this analysis should be acknowledged. Most included trials focused exclusively on COPD populations, limiting the generalizability of findings to other causes of hypercapnic respiratory failure. The predominance of single‐center studies and variability in methodological quality may also affect the reliability of the pooled estimates. Additionally, the pooled effects of HFNC on arterial blood gas parameters were less consistent across studies. Moreover, cardiac and vascular comorbidities—which have been shown to significantly reduce the likelihood of successful HFNC treatment [42, 43]—were not consistently reported, precluding subgroup analysis based on these important clinical factors. Large‐scale, multicenter RCTs are needed to further elucidate the comparative benefits of HFNC versus BiPAP across more diverse and heterogeneous patient populations.

## 5 Conclusion

In acute hypercapnic respiratory failure, BiPAP and HFNC achieve comparable outcomes, yet BiPAP confers slight improvements in gas exchange, acid–base balance, and respiratory rate. HFNC's enhanced comfort profile makes it an appealing alternative for patients who cannot tolerate BiPAP interfaces.

## Author Contributions

Y.H., N.L., and Y.Q. designed the meta‐analysis. Y.H. and N.L. searched for the articles, screened titles and abstracts, and extracted data. M.S., S.G., and Z.Z. performed statistical analysis and interpretation of data. M.S. and S.G. contributed to the conception of the study. Y.H. and N.L. drafted the manuscript, and Y.Q., M.S., S.G., and Z.Z. revised it for important intellectual content; all authors read and approved the final manuscript.

## Funding

This work was supported by grants from Suzhou Science and Education Strengthening Health Project (No. ZDXM2024005) and Suzhou Applied Basic Research (Medical and Health) Science and Technology Innovation Project (No. SYWD2024028).

## Ethics Statement

The authors have nothing to report.

## Consent

The authors have nothing to report.

## Conflicts of Interest

The authors declare no conflicts of interest.

## Supporting information


**Data S1:** Supporting information.

**Data S2:** Supporting information.

**Data S3:** Supporting information.

## Data Availability

The datasets used and/or analysed during the current study are available from the corresponding author on reasonable request.
