# Supplementary material for: High‐Flow Nasal Cannula in Hypercapnic Respiratory Failure: An Updated Systematic Review and Meta‐Analysis
Source: Clin Respir J. 2026 Jul 1;20(7):e70207. doi: 10.1111/crj.70207 (PMC13323174; doi:10.1111/crj.70207)
Supplement: Supplementary file 1 — Data S1: Supporting information. [file CRJ-20-e70207-s002.zip › Chinese_Article/Bian et al. 2022 “经鼻高流量氧疗与无创呼吸机在老年慢性阻塞性肺疾病急性加重期合并II型呼吸衰竭治疗中的应用效果.pdf]

经鼻高流量氧疗与无创呼吸机在老年慢性阻塞性肺疾病急性加重期合并Ⅱ型呼吸衰竭治疗中的应用效果

卞锋全　（泰兴市人民医院，江苏　泰兴　225400）

〔摘　要〕 **目的**　对比分析经鼻高流量氧疗（HFNC）与无创呼吸机在老年慢性阻塞性肺疾病（慢阻肺）急性加重期合并Ⅱ型呼吸衰竭（Ⅱ-RF）治疗中的应用效果。**方法**　选取 100 例慢阻肺急性加重期合并Ⅱ-RF 患者，依据随机数字表法分为 HFNC 组与无创呼吸机组各 50 例，观察两组临床指标（呼吸困难缓解时间、呼吸支持时间、住院时间），对比两组治疗前、治疗 24 h 血气指标〔动脉血二氧化碳分压（$PaCO_2$）、动脉血氧分压（PaO_2）、动脉血 pH 值、血氧饱和度（SpO_2）〕，治疗 24 h 时，采用 Kolcaba 研制的舒适状况量表（GCQ）评估两组治疗舒适度；统计两组并发症及终止治疗率情况。**结果**　两组呼吸困难缓解时间、呼吸支持时间、住院时间差异无统计学意义（$P>0.05$）；治疗 24 h，两组 $PaCO_2$ 低于治疗前，PaO_2、动脉血 pH 值、SpO_2 高于治疗前，差异有统计学意义（$P<0.05$）；但组间差异无统计学意义（$P>0.05$）；治疗 24 h，HFNC 组 GCQ 评分高于无创呼吸机组，差异有统计学意义（$P<0.05$）；HFNC 组并发症发生率、终止治疗率低于无创呼吸机组，差异有统计学意义（$P<0.05$）。**结论**　HFNC 与无创呼吸机均为治疗老年慢阻肺急性加重期合并Ⅱ-RF 患者的有效方法，可快速缓解患者呼吸困难症状，改善血气指标，缩短呼吸支持及住院时间，其中 HFNC 舒适度较高、治疗终止率较低，且并发生较少。

〔关键词〕 慢性阻塞性肺疾病；急性加重期；Ⅱ型呼吸衰竭；经鼻高流量氧疗；无创呼吸机

〔中图分类号〕 **R563**　〔文献标识码〕 **A**　〔文章编号〕 **1005-9202(2022)21-5223-04；doi：10.3969/j.issn.1005-9202.2022.21.020**

慢性阻塞性肺疾病（慢阻肺）多发于老年人群，病程较长，气流受限呈进行性发展趋势，且病情极易反复发作，引发Ⅱ型呼吸衰竭（Ⅱ-RF），致使患者出现呼吸困难、气短等症状，甚至威胁患者生命[1]。无创呼吸机是治疗老年慢阻肺急性加重期合并Ⅱ-RF 患者基础且有效的方法，可快速改善患者通气状况，减轻呼吸困难症状，缓解病情，但该治疗方式可引起人机对抗、口鼻干燥、胃胀气、面罩压伤、误吸等并发症，影响临床效果[2]。近年来，经鼻高流量氧疗在临床广泛应用，可对吸入气体进行加湿、加温处理，患者可自由呼吸、饮食，更加符合人体生理要求，且具有供氧浓度精确、可高浓度给氧、高送气流量的优势，可有效改善患者通气和供氧功能[3]。本研究拟对比分析上述两种治疗方式在老年慢阻肺急性加重期合并Ⅱ-RF 患者中的应用效果。

1　资料与方法

1.1　一般资料　选取 2018 年 7 月至 2021 年 6 月泰兴市人民医院 100 例慢阻肺急性加重期合并Ⅱ-RF 患者，依据随机数字表法分为 HFNC 组与无创呼吸机组各 50 例。纳入标准：（1）符合慢阻肺急性加重相关诊断标准[4]，且经肺功能、影像学检查确诊；（2）符合Ⅱ-RF 相关诊断标准[5]；（3）轻中度患者；（4）呼吸道结构正常；（5）血流动力学稳定；（6）患者或家属签署知情同意书。排除标准：（1）持续性气道分泌物排出困难；（2）呼吸暂停、心搏骤停导致窒息或意识丧失；（3）合并严重心律失常、心力衰竭；（4）反应迟钝、心率<50 次/min；（5）合并严重肝肾功能障碍；（6）无自主呼吸，不能配合经鼻高流量氧疗。HFNC 组男 29 例，女 21 例；年龄 60～79 岁，平均（67.35±3.12）岁；慢阻肺病程 2～13 年，平均（7.58±2.37）年；体重指数 18～25 kg/m²，平均（21.59±1.02）kg/m²；受教育程度：小学 29 例，初中、高中 18 例，高中以上 3 例。无创呼吸机组男 27 例，女 23 例；年龄 60～78 岁，平均（67.28±3.26）岁；慢阻肺病程 2～14 年，平均（7.63±2.41）年；体重指数 18～26 kg/m²，平均（21.64±1.10）kg/m²；受教育程度：小学 27 例，初中、高中 19 例，高中以上 4 例。两组一般资料差异无统计学意义（$P>0.05$），具有可比性。本研究经医院医学伦理委员会审核批准。

1.2　方法　两组均给予（1）抗感染：取 3.75 g 哌拉西林他唑巴坦（华北制药集团北元有限公司，国药准字 H20100062，规格：1.25 g）加入 20 ml 氯化钠注射液充分溶解后，加入 100 ml 浓度为 0.9% 的氯化钠注射液，静脉滴注，滴注时间≥30 min，每 8 h 一次或每 12 h 一次；（2）解痉平喘：取 200 mg 多索茶碱（BIOLOGICAL ITALIA LABORATORIES S.R.L，进口药品注册证号 H20170323，规格：10 ml∶0.1 g）加

第一作者：卞锋全（1973-），男，副主任医师，主要从事急诊急危重症的诊断和治疗研究。

入50 ml浓度为0.9%的氯化钠注射液稀释，缓慢静脉泵注，4.2 ml/h，每12 h一次；(3)祛痰：取4 mg溴己新（广州一品红制药有限公司，国药准字H20051532，规格：4 mg）加入100 ml浓度为5%的葡萄糖注射液稀释，静脉滴注，2次/d；(4)自主呼吸较慢时（如低于16次/min）给予兴奋呼吸：取9 mg洛贝林（上海禾丰制药有限公司，国药准字H31021342，规格：1 ml∶3 mg）+1.125 g尼可刹米（遂成药业股份有限公司，国药准字：H41021044，规格：1.5 ml∶0.375 g）加入50 ml浓度为0.9%的氯化钠注射液稀释，静脉泵注，4.2 ml/h，每12 h一次。(5)如气喘明显、双肺闻及明显哮鸣音提示气道痉挛或水肿明显时，加用40 mg甲泼尼龙（Pfizer Manufacturing Belgium N.V. 进口药品注册证号：H20170197，规格：40 mg）加入100 ml浓度为0.9%氯化钠注射液稀释，静脉滴注，1～2次/d。

1.2.1　HFNC组　采用经鼻高流量氧疗，采用沈阳迈思医疗科技有限公司生产的型号为HUMID-BM的高流量呼吸湿化治疗仪，依据患者个体情况选择合适规格的鼻塞，使用时将仪器加温至37℃，吸入氧浓度为30%，可按照患者病情适度调节氧气浓度，氧气流量：40～60 L/min，将患者血氧饱和度（SpO_2）维持在92%以上。每次通气治疗时间为8 h，间歇15～30 min。

1.2.2　无创呼吸机组　采用无创呼吸机治疗，采用德国万曼医疗器械有限公司生产的型号为VENTI-motion 2的无创呼吸机，呼吸机模式：S/T，采用口鼻面罩通气治疗，初始参数：吸气压力8～10 cmH_2O，呼气压力4 cmH_2O，呼吸频率12～16次/min，氧流量8～10 L/min，依据患者耐受程度适当调整上述参数。每次通气治疗时间为6 h，间歇15～30 min。

1.3　观察指标　(1)临床指标：记录两组呼吸困难缓解时间、呼吸支持时间、住院时间。(2)血气指标：采用血气分析仪（东莞键威医疗器械有限公司，DH-1830）监测两组治疗前、治疗24 h动脉血二氧化碳分压（$PaCO_2$）、动脉血氧分压（PaO_2）、动脉血pH值、SpO_2。(3)舒适度：治疗24 h，采用Kolcaba研制的舒适状况量表（GCQ）[6]评估两组治疗舒适度，该表包含4个维度，即社会、心理、环境及生理，共28个条目，每个条目采用4级评分法，总分112分，分值越高表明舒适度越佳。(4)并发症及终止治疗率：统计两组并发症（胃胀气、面罩压伤、口干）发生情况。

1.4　统计学方法　采用SPSS25.0统计学软件进行t检验、χ^2检验。

2　结　果

2.1　两组临床指标比较　两组呼吸困难缓解时间、呼吸支持时间、住院时间差异无统计学意义（P>0.05）。见表1。

表1　两组临床指标比较（$\bar{x}\pm s$，n=50）

组别	呼吸困难缓解时间(h)	呼吸支持时间(d)	住院时间(d)
HFNC组	7.20±1.48	8.13±1.62	15.26±1.87
无创呼吸机组	6.89±1.52	7.86±1.34	15.63±1.58
t/P值	1.033/0.304	0.908/0.366	1.069/0.288

2.2　两组治疗前、治疗24 h血气指标比较　治疗24 h，两组$PaCO_2$水平低于治疗前，PaO_2、动脉血pH值、SpO_2水平均高于治疗前，差异有统计学意义（P<0.05）；但组间比较差异无统计学意义（P>0.05）。见表2。

表2　两组治疗前、治疗24 h血气指标比较（$\bar{x}\pm s$，n=50）

时间	组别	$PaCO_2$(mmHg)	PaO_2(mmHg)	动脉血pH值	SpO_2(%)
治疗前	HFNC组	56.79±6.12	55.29±4.86	7.31±0.08	85.16±2.13
	无创呼吸机组	57.18±6.34	54.87±5.02	7.32±0.09	84.97±2.09
	t/P值	0.313/0.755	0.425/0.672	0.587/0.558	0.450/0.654
治疗24 h	HFNC组	49.06±2.35$^{1)}$	67.48±3.66$^{1)}$	7.36±0.04$^{1)}$	97.62±0.65$^{1)}$
	无创呼吸机组	48.74±3.16$^{1)}$	67.76±4.25$^{1)}$	7.37±0.03$^{1)}$	97.58±0.76$^{1)}$
	t/P值	0.574/0.567	0.353/0.725	1.414/0.161	0.283/0.778

与同组治疗前比较：1)P<0.05

2.3　两组舒适度比较　治疗24 h，HFNC组GCQ评分为(89.62±3.61)分，无创呼吸机组为(72.58±4.25)分，两组差异有统计学意义（t=21.606，P<0.05）。

2.4　两组并发症及终止治疗率比较　HFNC组并发症发生率〔2例（4.00%）；胃胀气、口干均1例〕低于无创呼吸机组〔9例（18.00%），其中胃胀气2例、面罩压伤4例、口干2例〕；终止治疗率〔1例

(2.00%)]低于无创呼吸机组[8 例(16.00%)],差异有统计学意义(χ^2 = 5.005、4.396,P = 0.025、0.036)。

3　讨　论

现阶段,慢阻肺的病因尚未完全明确,多数与吸烟、化学物质、有害颗粒吸入、呼吸道感染等因素有关[7]。气道阻塞、气流受限是老年慢阻肺急性加重期患者主要病理改变,可导致患者出现不同程度二氧化碳潴留、缺氧症状,引发Ⅱ-RF,危及患者生命。积极采取相关治疗措施,改善患者通气功能是治疗老年慢阻肺急性加重期合并Ⅱ-RF 患者的关键。

无创呼吸机是临床治疗老年慢阻肺急性加重期合并Ⅱ-RF 患者常用的机械通气方式,治疗过程中无需建立人工气道,具有简便、灵活的特点,易于被患者接受,治疗过程中采用面罩吸氧的方式可达到更高的吸入氧浓度,快速改善患者临床症状[8,9]。HFNC 吸氧治疗装置由鼻塞系统、专业加温加湿系统、高流量输出装置三大部分组成,其专业加温加湿系统可提供舒适的温度及湿度,可有效减少对鼻咽部刺激,保护气道纤毛黏液系统功能,有利于痰液稀释排出,增加患者舒适度[10,11]。本研究结果显示,HFNC 与无创呼吸机均可快速改善老年慢阻肺急性加重期合并Ⅱ-RF 患者呼吸困难症状,获得良好临床效果。

$PaCO_2$、PaO_2、动脉血 pH 值、SpO_2 是临床常见血气指标,可准确反映人体呼吸功能及酸碱平衡状态[12]。本研究结果提示 HFNC 在改善老年慢阻肺急性加重期合并Ⅱ-RF 患者血气指标方面效果与无创呼吸机相当。分析其原因,HFNC 具有稳定的供氧性能,可提供 21% ~ 100% 恒定氧浓度,同时其密闭导管可输送最大达 60 L/min 流量的空氧混合气体以产生轻度持续气道正压、冲刷减少呼气末残留在鼻咽部的含有较高浓度二氧化碳的呼出气体,增加呼气末肺容积,减少无效腔通气,提升通气功能,改善血气指标[13,14]。

本研究结果还提示与无创呼吸机相比,HFNC 治疗老年慢阻肺急性加重期合并Ⅱ-RF 患者可减少并发症,降低终止治疗率。究其原因,无创呼吸机治疗过程中所使用的面罩可增加患者拘束感,对于有幽闭恐惧症患者会导致恐惧感,由于频繁咳嗽、不会配合及灵敏度设置不合理可导致明显的人机对抗,增加患者的恐惧和抵制情绪,甚至对鼻翼及鼻部周围皮肤产生压迫损伤,面罩还可对患者进食、饮水产生直接影响,增加胃胀气发生率[15],严重的导致治疗中止。而 HFNC 吸氧装置保持湿化用水的密闭性、加温加湿的连续性,有效避免气道水分流失、呼吸道分泌物黏稠聚积,增加肺部顺应性,提高治疗舒适度[16]。HFNC 利用鼻塞实施氧疗,无需面部加压,患者可自由呼吸、说话,不影响患者进食、饮水,从而减少相关并发症,且具有良好舒适性。此外,HFNC 治疗过程中对吸入气体充分加温、加湿,减少传统吸氧装置导致的口鼻腔干燥及气道黏膜损伤[17,18],能有效促进痰液排出,减轻气道阻力,改善通气和氧合功能,降低终止治疗率。

综上,HFNC 与无创呼吸机均为治疗老年慢阻肺急性加重期合并Ⅱ-RF 患者的有效方法,可快速缓解患者呼吸困难症状,改善血气指标,缩短呼吸支持及住院时间,其中 HFNC 舒适度较高,治疗终止率较低,且并发生较少。

4　参考文献

1　Xiao W,Du LY,Mao B,*et al*. Endotype-driven prediction of acute exacerbations in chronic obstructive pulmonary disease (End AECOPD):protocol for a prospective cohort study[J]. BMJ Open,2019;9(11):e034592.

2　Marchioni A,Tonelli R,Fantini R,*et al*. Respiratory mechanics and diaphragmatic dysfunction in COPD patients who failed non-invasive mechanical ventilation[J]. Int J Chron Obstruct Pulmon Dis,2019;22(14):2575-85.

3　Tung LF,Shen SY,Shih HH,*et al*. Effect of high-flow nasal therapy during early pulmonary rehabilitation in patients with severe AECOPD:a randomized controlled study[J]. Respir Res,2020;21(1):84.

4　慢性阻塞性肺疾病急性加重(AECOPD)诊治专家组. 慢性阻塞性肺疾病急性加重(AECOPD)诊治中国专家共识(2017 年更新版)[J]. 国际呼吸杂志,2017;37(14):1041-57.

5　严重急性低氧性呼吸衰竭急诊治疗专家共识组. 严重急性低氧性呼吸衰竭急诊治疗专家共识[J]. 中华急诊医学杂志,2018;27(8):844-9.

6　张莉,何利,孟珊珊,等. 中文版制动舒适度问卷在膝关节置换术后患者中应用的信效度分析[J]. 中华现代护理杂志,2018;24(6):647-51.

7　Chen C,Liu X,Wang X,*et al*. Risk of temperature,humidity and concentrations of air pollutants on the hospitalization of AECOPD[J]. PLoS One,2019;14(11):e0225307.

8　Francesca R,Costanza C,Matteo S,*et al*. Surfactant replacement therapy in combination with different non-invasive ventilation techniques in spontaneously-breathing, surfactant-depleted adult rabbits[J]. PLoS One,2018;13(7):e0200542.

9　Suri TM,Esquinas A,Hadda V,*et al*. HVNI vs NIPPV in the treatment of acute decompensated heart failure:is acute stabilization enough[J]? Am J Emerg Med,2019;37(8):1588-9.

10　陈红梅,刘利. 经鼻高流量湿化氧疗对 AECOPD 患者血气分析、再插管率及舒适度的影响[J]. 昆明医科大学学报,2019;40(7):116-9.

11 张京臣,吴逢选,孟琳琳,等. 慢性阻塞性肺疾病患者拔管后序贯经鼻高流量氧疗的效果及安全性〔J〕. 中华医学杂志,2018;98(2):109-12.

12 刘林林,栾英,肖凌,等. COPD 急性加重期患者血清 PCT、hs-CRP、血气指标变化及其与预后的相关性〔J〕. 新疆医科大学学报,2019;42(9):1180-3,1188.

13 Bae SH,Han M,Kim C,*et al.* High-flow nasal cannula oxygen therapy can be effective for patients in acute hypoxemic respiratory failure with hypercapnia: a retrospective, propensity score-matched cohort study〔J〕. J Korean Med Sci,2020;35(10):e67.

14 Zhu Y,Yin H,Zhang R,*et al.* High-flow nasal cannula oxygen therapy versus conventional oxygen therapy in patients after planned extubation: a systematic review and meta-analysis〔J〕. Crit Care,2019;23(1):180.

15 胡述立,汤浩,范学朋. 经鼻高流量氧疗与无创正压通气在不同 APACHE Ⅱ评分老年 AECOPD 患者中拔管后的治疗效果研究〔J〕. 中国全科医学,2018;21(15):1790-5.

16 郑泓斌,张淇钏. 经鼻高流量氧疗与无创正压通气在慢性阻塞性肺疾病急性加重期治疗中的效果比较〔J〕. 广东医学,2019;40(10):1443-6.

17 Huang HB,Peng JM,Weng L,*et al.* High-flow oxygen therapy in immunocompromised patients with acute respiratory failure: a review and meta-analysis〔J〕. J Crit Care,2018;32(43):300-5.

18 谈定玉,凌冰玉,孙家艳,等. 经鼻高流量氧疗与无创正压通气比较治疗慢性阻塞性肺疾病合并中度呼吸衰竭的观察性队列研究〔J〕. 中华急诊医学杂志,2018;27(4):361-6.

〔2022-03-25 修回〕

（编辑　滕欣航）

BiPAP 无创通气联合莫西沙星溶液雾化吸入治疗老年慢性阻塞性肺疾病合并Ⅱ型呼吸衰竭的疗效

任英杰　汪桂青　赵美英　李平　（郑州大学附属郑州中心医院老年医学科,河南　郑州　450000）

〔摘　要〕 **目的**　探讨老年慢性阻塞性肺疾病(慢阻肺)合并Ⅱ型呼吸衰竭(RF)采用 BiPAP 无创通气(NIV)联合莫西沙星溶液雾化吸入治疗的疗效。**方法**　选取 120 例慢阻肺合并 RF 患者,根据入院单号将其分为对照组和观察组各 60 例。两组均采用常规治疗,在此基础上,对照组采用低浓度氧疗联合莫西沙星溶液雾化吸入治疗,观察组采用 BiPAP NIV 联合莫西沙星溶液雾化吸入治疗。比较两组治疗 1 w 临床疗效,治疗前、治疗 1 w 血气指标〔动脉二氧化碳分压($PaCO_2$)、动脉血氧分压(PaO_2)、动脉血氧饱和度(SaO_2)〕、肺功能〔第 1 秒用力呼气容积(FEV1)、FEV1 占用力肺活量(FVC)的百分比(FEV1/FVC)〕。**结果**　治疗 1 w,观察组临床总有效率显著高于对照组($P<0.05$);治疗 1 w,两组 $PaCO_2$ 水平显著低于治疗前,SaO_2、PaO_2 水平显著高于治疗前,且观察组 $PaCO_2$ 水平显著低于对照组,SaO_2、PaO_2 水平显著高于对照组($P<0.05$,$P<0.001$);治疗 1 w,两组 FEV1、FEV1/FVC 水平显著高于治疗前,且观察组显著高于对照组($P<0.05$)。**结论**　BiPAP NIV 联合莫西沙星溶液雾化吸入治疗老年慢阻肺合并Ⅱ型 RF 效果显著,可改善患者氧合,提高肺功能。

〔关键词〕 慢性阻塞性肺疾病;呼吸衰竭;无创通气;莫西沙星溶液;肺功能

〔中图分类号〕 R563.8　〔文献标识码〕 A　〔文章编号〕 1005-9202(2022)21-5226-03;doi:10.3969/j.issn.1005-9202.2022.21.021

慢性阻塞性肺疾病(慢阻肺)受有害气体、异常炎症反应影响,可进一步引发呼吸衰竭(RF),致残率、病死率较高[1]。目前,临床治疗慢阻肺合并 RF 患者多采用抗感染治疗,以免患者出现肺部感染。莫西沙星抗菌活性较强,在多种疾病抗感染治疗中应用较为广泛[2]。而针对合并 RF 患者,临床常采用氧疗,以维持患者呼吸系统运行,避免病情加重。但因老年人群多合并基础疾病,一旦合并 RF,病情进展迅速,单纯氧疗可能无法满足患者的呼吸需求[3]。因此,为促进患者病情改善,探寻其他有效方案尤为重要。无创通气(NIV)是一种由呼吸机完成通气辅助的人工通气方式,具有无创、舒适度高等优势,降低患者插管率[4]。本研究旨在探讨 BiPAP NIV 联合莫西沙星溶液雾化吸入治疗老年慢阻肺合并Ⅱ型 RF 疗效。

1　资料与方法

1.1　一般资料　选取郑州大学附属郑州中心医院 2019 年 10 月至 2020 年 10 月收治的 120 例慢阻肺合并 RF 患者,根据入院单号将其分为对照组和观察组各 60 例。对照组男 38 例,女 22 例;年龄 62～78 岁,平均(70.02±6.84)岁;病程 2～10 年,平均(6.28±1.36)年;其中有吸烟史 18 例,有饮酒史 16 例。观察组男 33 例,女 27 例;年龄 61～77 岁,平均(69.26±6.37)岁;病程 2～12 年,平均(6.95±1.71)年;其中有吸烟史 23 例,有饮酒史 12 例。两组基线资料差异无统计学意义($P>0.05$),具有可比性。纳

第一作者:任英杰(1976-),女,硕士,副主任医师,主要从事老年呼吸与危重症研究。
