# Supplementary material for: High‐Flow Nasal Cannula in Hypercapnic Respiratory Failure: An Updated Systematic Review and Meta‐Analysis
Source: Clin Respir J. 2026 Jul 1;20(7):e70207. doi: 10.1111/crj.70207 (PMC13323174; doi:10.1111/crj.70207)
Supplement: Supplementary file 1 — Data S1: Supporting information. [file CRJ-20-e70207-s002.zip › Chinese_Article/Liu et al. 2019 ''高流量氧疗和无创通气在COPD伴轻度+II型呼吸衰竭患者中的应用比较.pdf]

文章编号:1007－4287(2019)09－1581－02

高流量氧疗和无创通气在COPD伴轻度Ⅱ型呼吸衰竭患者中的应用比较

刘晓娟[1],曹大伟[2],张新日[2*]

(1.山西医科大学,山西 太原 030600;2.山西医科大学第一医院,山西 太原 030001)

慢性阻塞性肺疾病(COPD)是一种常见的以持续气流受限为特征的慢性疾病,常导致呼吸衰竭。COPD在我国北方尤其多发,严重危害中老年人群的健康[1]。纠正低氧血症是COPD治疗的根本。无创通气(NIV)是治疗呼吸衰竭的经典手段,2019GOLD指南统计NIV在治疗COPD的成功率方面可达到80%-85%[2],然而实际中存在NIV禁忌证或不耐受治疗的患者仍较多,制约其进一步应用。经鼻高流量氧疗(HFNC)是一种通过无需密封的导管,经鼻输入经过加温加湿的高流量混合气体的新型无创通气氧疗方式。2000年开始应用于临床,在新生儿及儿童呼吸功能不全患者中已经得到广泛的使用,近年来才逐渐应用于成人患者,已被证实对I型呼吸衰竭有良好的效果[3]。本研究拟通过对比HFNC和NIV治疗COPD合并轻度Ⅱ型呼吸衰竭患者,来探讨HFNC对COPD合并呼吸衰竭预后的影响。

1 资料与方法

1.1 病例资料

选取2017年3月至2018年12月因COPD合并Ⅱ型呼吸衰竭而入住我科的患者60例。其中男性31例,女性29例,年龄55-82岁。(1)纳入标准:①符合慢性阻塞性肺部疾病全球倡议[2]中COPD定义;②年龄>55岁;③氧分压<50 mmHg,二氧化碳分压50-70 mmHg。

(2)病例排除标准:①治疗过程中病情进行性加重或者放弃治疗患者;②除呼吸系统外严重器官功能异常患者;③合并其它疾病导致意识障碍不能配合高流量氧疗和无创呼吸机患者。

将符合纳入标准的患者随机分成经鼻高流量氧疗(HFNC,H)组和无创正压通气(NIV,N)组,其中每组30例患者。各组之间的自然状况、治疗前基本情况无统计学差异(表1)。

* 通讯作者

万方数据

表1 两组患者的一般资料比较

组别	例数(n)	性别	年龄	氧分压	二氧化碳分压
高流量组(H)	30	16/14	66±9.36	48±4.36	65±5.28
无创组(N)	30	15/15	67±9.78	47±4.25	65±5.43

两组间比较,各指标 $P>0.05$

1.2 观察指标

两组患者均给予COPD的基础治疗,即抗感染、化痰、维持内环境稳定、保护脏器功能等。分别评估患者治疗前心率(HR)、平均动脉压(MAP)、动脉血氧分压(PaO_2)、二氧化碳分压($PaCO_2$)、乳酸(Lac),氧合指数(PaO_2/FiO_2),患者接受治疗后4 h、12 h的以上指标变化。

1.3 治疗方法

高流量氧疗组(H组):应用经鼻高流量吸氧仪器与鼻塞导管(AIRVO$_2$,新西兰费雪派克)进行通气,初始设置流量为20-40 L/min,温度37℃,FiO_2:30%-60%,以使目标SPO_2>90%,治疗过程中逐步调整。

无创通气组(N组):应用无创呼吸机接口鼻面罩(V60,荷兰飞利浦伟康)进行正压通气,S/T通气模式,FiO_2:30%-60%,吸气时间0.8-1.2 S,初始设置吸气压(IPAP)为10-12 cm H_2O,呼气压(EPAP)为4-6 cm H_2O,吸呼比1:(1.5-2.0),压力上升时间0.5-1.0 s,随时调整参数使目标达到SPO_2>90%。

2 结果

两组患者HR、MAP、PaO_2、$PaCO_2$、Lac、PaO_2/FiO_2治疗后均较治疗前明显好转,且治疗12 h时的临床指标优于治疗4 h时。可是在心率的下降方面,H组12小时较N组改善差异有显著性;动脉血氧分压方面,在4小时H组疗效较N组差异有显著性,到12小时点,两者疗效接近;在二氧化碳分压下降方面,N组较H组在两个治疗时间点都有优势,差异有显著性。在平均动脉压、乳酸和氧合指数方

面，两组比较差异无显著性（表 2）。在患者治疗中断方面，H 组明显较 N 组数量较少（表 3）。

表 2　两组治疗前后及不同时间点各项指标变化

指标	HFNC 组			NIV 组		
	治疗前	治疗 4 h	治疗 12 h	治疗前	治疗 4 h	治疗 12 h
HR	115.02±10.24	100.54±7.61*	83.27±5.38**#	116.35±9.82	99.34±6.52*	90.36±5.78**
MAP	105.53±6.75	93.58±5.27*	88.26±4.23**	106.63±6.19	95.86±5.62*	91.57±4.32**
PaO_2	48.58±4.36	92.72±5.13*#	111.53±6.47**	47.35±4.25	85.61±5.76*	109.62±6.98**
$PaCO_2$	65.43±5.28	55.83±5.12*	48.74±4.10**	65.37±5.43	51.96±5.39*#	42.61±4.35**#
Lac	5.24±1.02	3.65±0.64*	2.30±0.55**	5.47±1.41	3.24±0.71*	2.76±0.48**
PaO_2/FiO_2	95.21±5.64	215.68±6.41*	360.31±7.50**	96.38±5.90	211.20±6.98*	350.28±7.63**

两组治疗 4 h 与治疗前比较，* $P<0.05$；两组治疗 12 h 与 4 h 比较，** $P<0.05$；HR，12 h 时 H 组与 N 组比较，# $P<0.05$；PaO_2，4 h 时 H 组与 N 组比较，# $P<0.05$；$PaCO_2$，4 h 和 12 h 时 N 组与 H 组比较，# $P<0.05$。

表 3　两组治疗中断事件的比较

	中断例数	中断次数
高流量组（H）	2	3
无创组（N）	6	10

两组间比较，H 组例数与次数均较 N 组少

3　讨论

无创通气自诞生以来，已经越来越多用于治疗各种病因导致的呼吸衰竭，尤其是 COPD 及急性肺水肿患者，其通过恒定的容量或压力给予患者通气，并可保留自主呼吸，流速随之改变。大量的研究资料证实，无创辅助通气可降低患者有创气管插管率，减少感染并发症的发生，减少 ICU 停留时间及总住院时间，降低总体治疗费用[4]。目前无创通气的实施主要是通过口鼻面罩和鼻罩，其中患者经常出现不耐受的原因包括面罩过紧、头带不适以及气流致呼吸障碍、饮水进食受限等，因此患者的舒适度及耐受性成为制约无创通气的关键因素。本研究中，NIV 组患者二氧化碳分压下降较理想，但中断治疗例数较多，优缺点同时存在。

高流量氧疗通过提供恒定流速的气体，改善患者的呼吸困难症状，纠正低氧血症，而气道压随患者呼吸改变。HFNC 提供的加温加湿气流，避免了干冷气体给患者带来的不适，可降低气道的敏感度，减轻黏膜干燥程度，并促进气道分泌物的廓清。高流量的气体减少了呼吸道解剖死腔，满足了呼吸困难患者对吸气流量的需求，并减少吸气阻力，有效的缓解了呼吸肌疲劳[5]。其恒定的流速可以产生低水平的呼气末正压（4-10 cmH_2O），有助于抵消内源性呼气末正压的影响，以改善氧换气功能，提高氧合能力[6]。并且，患者佩戴舒适，除少数的鼻部解剖结构异常的患者，均可以长时间佩戴鼻塞，且不影响进食及语言功能。本研究中，HFNC 组在短时内改善氧合，长时降低心率效果好，可能和患者舒适度与耐受性更佳有关。

根据研究结果，HFNC 与 NIV 短期内均可明显改善 COPD 合并轻度Ⅱ型呼吸衰竭患者的呼吸困难，改善氧合。尽管在降低二氧化碳分压方面，HFNC 与 NIV 相比还有一定的差距，但考虑到在提升氧分压方面，HFNC 并未处于劣势，在舒适度和依从性更佳，并且操作简便，成本较低，其可作为 COPD 致轻度Ⅱ型呼吸衰竭患者氧疗的一种选择。

参考文献：

[1]Global Initiative for Chronic Obstructive Lung Disease. Global Initiative for Chronic Obstructive Lung Disease（2013）［EB/OL］.［2016-5-19］. http://www.goldcopd.com.

[2]Shen YC，Chen L，Wen FQ. Retation of 2019 Global Strategy for the Diagnosis，Management and Prevention of chronic Obstructive Pulmonary Disease[J]. Zhonghua Yi Xue Za Zhi，2018，98(48)：3913.

[3]岳伟岗，张志刚，张彩云，等. 经鼻高流量氧疗对呼吸衰竭患者疗效的 Meta 分析[J]. 中华危重病急救医学，2017，29(5)：396.

[4]米　崧，张黎明. 慢性阻塞性肺疾病患者无创通气时吸氧方式对氧分压及二氧化碳分压的影响[J]. 中华结核和呼吸杂志，2017，40(4)：267.

[5]远青钊，罗　琴，王在义. 经鼻高流量加温湿化吸氧治疗在慢性阻塞性肺病合并呼吸衰竭患者中应用的疗效观察[J]. 新疆医科大学学报，2018，41(5)：556.

[6]刘景刚，杨圣强，袁继印，等. 经鼻高流量氧疗序贯治疗慢性阻塞性肺疾病机械通气患者的临床研究[J]. 中华急诊医学杂志，28(4)：459.

（收稿日期：2019－03－13）

万方数据
