# Supplementary material for: High‐Flow Nasal Cannula in Hypercapnic Respiratory Failure: An Updated Systematic Review and Meta‐Analysis
Source: Clin Respir J. 2026 Jul 1;20(7):e70207. doi: 10.1111/crj.70207 (PMC13323174; doi:10.1111/crj.70207)
Supplement: Supplementary file 1 — Data S1: Supporting information. [file CRJ-20-e70207-s002.zip › Chinese_Article/Tang Y et al. 2023 ''经鼻高流量氧疗对AECOPD合并II型呼吸衰竭患者的疗效评价.pdf]

· 论著 ·

经鼻高流量氧疗对 AECOPD 合并Ⅱ型呼吸衰竭患者的疗效评价

唐艳平 兰福全

北京四季青医院内科，北京 100097

通信作者：唐艳平，Email：13911295821@139.com

【摘要】 **目的** 观察经鼻高流量氧疗（HFNC）与无创机械通气对慢性阻塞性肺疾病急性加重期（AECOPD）Ⅱ型呼吸衰竭（呼衰）患者的临床疗效。**方法** 选择2019年9月至2021年12月北京四季青医院内科收治的84例AECOPD合并Ⅱ型呼衰患者作研究对象。按随机原则将患者分为观察组和对照组，每组42例。两组患者均给予常规治疗，观察组给予HFNC，对照组给予无创机械通气。比较两组患者临床疗效的差异，并观察治疗前和治疗7 d后两组患者心率（HR）、呼吸频率（RR）、动脉血气分析指标、肺功能，以及并发症发生情况和气管插管率、病死率的变化。**结果** 观察组总有效率明显高于对照组〔88.10%（37/42）比69.05%（29/42），$P<0.05$〕。两组治疗前HR、RR、动脉血气分析指标〔动脉血氧分压（PaO_2）、动脉血二氧化碳分压（$PaCO_2$）、动脉血氧饱和度（SaO_2）、pH值〕、肺功能指标〔1秒用力呼气容积（FEV1）、用力肺活量（FVC）、FEV1/FVC〕比较差异均无统计学意义，治疗后HR、RR、$PaCO_2$均较治疗前明显降低，PaO_2、SaO_2、pH值、FEV1、FVC、FEV1/FVC均较治疗前明显升高，且治疗后观察组HR、RR、$PaCO_2$均明显低于对照组〔HR（次/min）：90.14±5.66比93.57±4.77，RR（次/min）：21.90±4.88比22.45±4.31，$PaCO_2$（mmHg，1 mmHg≈0.133 kPa）：60.02±5.93比62.83±6.29〕，PaO_2、SaO_2、pH值、FEV1、FVC、FEV1/FVC均明显高于对照组〔PaO_2（mmHg）：80.70±7.81比76.21±8.23，SaO_2：0.94±0.04比0.92±0.04，pH值：7.36±0.05比7.32±0.06，FEV1（L）：1.73±0.41比1.53±0.35，FVC（L）：2.80±0.48比2.57±0.43，FEV1/FVC：（61.98±5.67）%比（59.14±5.34）%，均$P<0.05$〕。观察组咽干〔4.8%（2/42）比19.0%（8/42）〕、面部压伤〔0%（0/42）比14.3%（6/42）〕、腹胀〔9.5%（4/42）比26.2%（11/42）〕等并发症发生率均较对照组明显降低（均$P<0.05$）。观察组和对照组气管插管率、病死率比较差异均无统计学意义〔11.90%（5/42）比26.19%（11/42）和4.76%（2/42）比7.14%（3/42），均$P>0.05$〕。**结论** 对AECOPD合并Ⅱ型呼衰患者采用HFNC疗效显著，且并发症少，安全性高。

【关键词】 经鼻高流量吸氧； 无创机械通气； 慢性阻塞性肺疾病； 呼吸衰竭

DOI：10.3969/j.issn.1008-9691.2023.05.007

Evaluation of the efficacy of high-flow nasal cannula oxygen therapy in acute exacerbation of chronic obstructive pulmonary disease complicated with type Ⅱ respiratory failure

Tang Yanping, Lan Fuquan

Department of Internal Medicine, Beijing Sijiqing Hospital, Beijing 100097, China

Corresponding author: Tang Yanping, Email: 13911295821@139.com

【**Abstract**】 **Objective** To observe the clinical efficacy of high-flow nasal cannula oxygen therapy (HFNC) and non-invasive mechanical ventilation in patients with type Ⅱ respiratory failure during acute exacerbation of chronic obstructive pulmonary disease (AECOPD). **Methods** A total of 84 patients with AECOPD combined with type Ⅱ respiratory failure admitted to the department of internal medicine of Beijing Sijiqing Hospital from September 2019 to December 2021 were selected as the study objects. The patients were randomly divided into observation group and control group, with 42 cases in each group. Both groups were given conventional treatment, the observation group was given HFNC, and the control group was given non-invasive mechanical ventilation. The difference of clinical efficacy between the two groups was compared, and the changes of heart rate (HR), respiratory rate (RR), arterial blood gas analysis index, lung function before and 7 days after treatment, complications and tracheal intubation rate, mortality were observed in the two groups. **Results** The total effective rate of the observation group was higher than that of the control group [88.10% (37/42) vs. 69.05% (29/42), $P < 0.05$]. Before treatment, HR, RR, arterial blood gas analysis indexes [arterial partial pressure of oxygen (PaO_2), arterial partial pressure of carbon dioxide ($PaCO_2$), arterial oxygen saturation (SaO_2), pH value], pulmonary function indexes [forced expiratory volume in one second (FEV1), forced vital capacity (FVC), FEV1/FVC] of the two groups were no statistically significant. After treatment, HR, RR and $PaCO_2$ were significantly lower than before treatment, while PaO_2, SaO_2, pH, FEV1, FVC and FEV1/FVC were significantly higher than before treatment. After treatment, HR, RR and $PaCO_2$ in the observation group were significantly lower than those in the control group [HR (bpm): 90.14±5.66 vs. 93.57±4.77, RR (times/min): 21.90±4.88 vs. 22.45±4.31, $PaCO_2$ (mmHg, 1 mmHg≈0.133 kPa): 60.02±5.93 vs. 62.83±6.29], PaO_2, SaO_2, pH, FEV1, FVC, FEV1/FVC were significantly higher than the control group [PaO_2 (mmHg): 80.70±7.81 vs. 76.21±8.23, SaO_2: 0.94±0.04 vs. 0.92±0.04, pH value: 7.36±0.05 vs. 7.32±0.06, FEV1 (L): 1.73±0.41 vs. 1.53±0.35, FVC (L): 2.80±0.48 vs. 2.57±0.43, FEV1/FVC: (61.98±5.67)% vs. (59.14±5.34)%, all $P < 0.05$]. The incidence of complications such as of dry throat [4.8%

(2/42) vs. 19.0% (8/42)], facial compression injury [0% (0/42) vs. 14.3% (6/42)], abdominal distension [9.5% (4/42) vs. 26.2% (11/42)] in the observation group were significantly lower than those in the control group (all $P < 0.05$). There was no significant difference in the rate of tracheal intubation and mortality between the observation group and the control group [11.90% (5/42) vs. 26.19% (11/42) and 4.76% (2/42) vs. 7.14% (3/42), both $P > 0.05$]. **Conclusion** HFNC is effective in treating AECOPD patients with type Ⅱ respiratory failure, with fewer complications and high safety.

【Key words】 High-flow nasal cannula oxygen therapy; Non-invasive mechanical ventilation; Chronic obstructive pulmonary disease; Respiratory failure

DOI: 10.3969/j.issn.1008-9691.2023.05.007

慢性阻塞性肺疾病(chronic obstructive pulmonary disease, COPD)是呼吸系统疾病中的常见病,以持续存在的呼吸道症状和气流不可逆地受限为特征。2018 年中国成人肺部健康研究(the China Pulmonary Health Study, CPHS)公布的流行病学调查结果显示,我国 40 岁以上人群 COPD 的发病率为 13.7% [1]。随着病情的进展,可出现呼吸衰竭(呼衰)、肺心病、肺性脑病等严重并发症,病死率较高[2]。COPD 急性加重期(acute exacerbation of COPD, AECOPD)合并呼衰是 COPD 患者死亡的重要原因[3],而此时的治疗除给予抗感染、祛痰、解除支气管痉挛等常规药物外,如何能在极短的时间内恢复患者有效通气量是治疗 AECOPD 的关键所在。

过去针对 AECOPD 多采用无创机械通气或持续低流量鼻导管吸氧的方式进行治疗。无创机械通气疗效好,但并发症也比较多;同时部分无创机械通气患者会因头面部压迫感和皮肤受压的疼痛感而产生焦虑及恐惧情绪,导致患者不能良好地配合,频繁摘除面罩,中断通气,影响通气疗效,从而影响疗效[4]。鼻导管吸氧技术简单,操作方便,但无法长时间提供稳定的高流量供氧,对重症患者通气的改善疗效较差,且作为湿化液的灭菌蒸馏水会引起患者鼻咽部不适[5]。近年来,有学者致力于研究如何改善和优化 AECOPD 患者的氧疗技术及临床疗效,更新并健全氧疗理念[6]。经鼻高流量氧疗(high-flow nasal cannula oxygen therapy, HFNC)作为一种相对新型的氧疗技术,与无创机械通气相比,具有独特的生理作用,不仅对患者进食、说话等方面的影响小,而且降低了并发症的发生率,提高了 AECOPD 患者氧疗期间的舒适度和耐受性,对维持患者稳定的血氧饱和度及呼气末正压(positive end-expiratory pressure, PEEP)方面更有利;并且可改善肺顺应性,降低气道阻力,增加功能残气量,防止肺泡塌陷,促使塌陷的肺泡复张,从而提高患者的通气状态[7]。因此,本研究通过比较 HFNC 与无创机械通气对 AECOPD 合并Ⅱ型呼衰患者疗效的差异,以期为 AECOPD 的治疗提供参考。

1 资料与方法

1.1 研究对象:选择 2019 年 9 月至 2021 年 12 月本院内科收治的 84 例 AECOPD 合并Ⅱ型呼衰患者作为研究对象。

1.1.1 纳入标准:① 符合 COPD 合并Ⅱ型呼衰的诊断标准[8];② 年龄>18 岁;③ 无心、肝、肾等重要器官功能衰竭。

1.1.2 排除标准:① 病情危重需进行有创通气;② 存在严重的血流动力学不稳定、大咯血、面部外伤或感染等无创通气、HFNC 禁忌证;③ 反复误吸或持续呕吐;④ 有严重精神异常及其他原因不能配合;⑤ 患有恶性肿瘤或机体免疫力下降。

1.1.3 伦理学:本研究符合医学伦理学标准,并经本院科研部门审核批准(审批号:2019-06),对患者采取的检查和治疗均获得患者或家属的知情同意。

1.2 研究分组及一般资料(表 1):将患者按随机原则分为观察组和对照组,每组 42 例。两组患者性别、年龄、病程比较差异均无统计学意义(均 $P>0.05$)。

表 1　不同治疗方法两组 AECOPD Ⅱ型呼衰患者一般资料的比较

组别	例数(例)	性别(例)		年龄(岁,$\bar{x}\pm s$)	病程(年,$\bar{x}\pm s$)
		男性	女性		
对照组	42	22	20	65.05±6.98	8.69±1.84
观察组	42	23	19	64.68±7.27	8.35±1.94

注:对照组给予无创机械通气,观察组行 HFNC

1.3 治疗方法:依据《COPD 临床诊治指南(2013 年修订版)》[9]给予患者常规对症治疗 7 d,包括控制感染、解痉平喘、止咳排痰、维持水和电解质及酸碱平衡、营养支持等治疗,指导患者排痰、协助变换体位,给予合理饮食、心理疏导、疾病知识健康宣教等。

1.3.1 对照组:给予无创机械通气。采用飞利浦 V35 型无创呼吸机辅助通气,基本参数:模式为双水平气道正压通气,吸气气道正压(inspiratory positive airway pressure, IPAP)14~25 cmH_2O($1 cmH_2O \approx 0.098$ kPa),PEEP 4~10 cmH_2O,呼吸频率 16~18 次/min,潮气量 7~10 mL/kg,吸入氧浓度(fraction of inspiration oxygen, FiO_2)0.35~0.50,氧流量 3~7 L/min,动脉

血氧饱和度（arterial oxygen saturation，SaO_2）≥0.92，治疗过程中根据患者的呼吸频率（respiratory rate，RR）、心率（heart rate，HR）、血气指标等实际情况调整参数（在患者耐受的情况下尽量上机）。

1.3.2 观察组：给予 HFNC。采用新西兰 Fisher& Paykel 公司生产的 AIRVO 呼吸湿化治疗仪及配套的 Optiflow 双腔鼻导管，参数设置：湿化温度控制在 37 ℃，吸入气体的流量 35～60 L/min，FiO_2 维持在 0.30～0.50，相对湿度 100%，SaO_2 维持在≥0.92。

1.4 观察指标及方法：① 观察两组患者治疗 7 d 后的临床疗效：显效为治疗后患者精神状态明显好转，胸闷、气促、发绀等症状缓解，HR 下降至 100 次 /min 以下，动脉血气分析示：pH 值 7.35～7.45，动脉血氧分压（arterial partial pressure of oxygen，PaO_2）＞60 mmHg（1 mmHg≈0.133 kPa），动脉血二氧化碳分压（arterial partial pressure of carbon dioxide，$PaCO_2$）＜60 mmHg，血流动力学稳定；有效为治疗后患者精神状态较前好转，胸闷、气促、发绀等症状好转，HR 较前下降 10～20 次 /min，动脉血气分析示：PaO_2＞60 mmHg，$PaCO_2$＜60 mmHg 或较前下降 10～20 mmHg，血流动力学稳定；无效为治疗后患者胸闷、气促、发绀等症状无明显改善或较前加重，意识变差，PaO_2 较前无变化或持续下降，$PaCO_2$ 较前无变化或持续升高，或需要气管插管行有创机械通气及死亡。总有效率＝（显效＋有效）/ 总例数 ×100%；② 记录两组患者治疗前及治疗 7 d 后的 RR、HR、血气分析指标（PaO_2、$PaCO_2$、SaO_2 及 pH 值）；③ 观察两组患者治疗前后肺功能指标变化：1 秒用力呼气容积（forced expiratory volume in one second，FEV1）、用力肺活量（forced vital capacity，FVC）、FEV1/FVC，治疗无效需插管上机者记录其上机前血气分析指标，以及出现并发症（如咽干、面部压伤、腹胀）的发生情况、30 d 内气管插管率和病死率。

1.5 统计学方法：使用 SPSS 22.0 统计软件分析数据。符合正态分布的计量数据以均数 ± 标准差（$\bar{x} \pm s$）表示，采用 t 检验；计数资料以例（率）表示，采用 χ^2 检验。$P<0.05$ 为差异有统计学意义。

2 结 果

2.1 两组临床疗效的比较（表 2）：观察组总有效率明显高于对照组（$\chi^2=4.525$，$P=0.033$）。

表 2 不同治疗方法两组 AECOPD Ⅱ型呼衰患者临床疗效的比较

组别	例数（例）	临床疗效（例） 显效	有效	无效	总有效率〔%（例）〕
对照组	42	9	20	13	69.05（29）
观察组	42	13	24	5	88.10（37）[a]

注：对照组给予无创机械通气，观察组行 HFNC；与对照组比较，[a]$P<0.05$

2.2 两组治疗前后 HR、RR 及血气分析指标的比较（表 3）：两组治疗前 HR、RR、血气分析指标比较差异均无统计学意义（均 $P>0.05$），治疗后 HR、RR、$PaCO_2$ 均较治疗前明显降低，PaO_2、SaO_2、pH 值较治疗前明显升高，且以观察组的变化更显著（均 $P<0.05$）。两组治疗后 RR 比较差异无统计学意义（$P>0.05$）。

2.3 两组治疗后肺功能指标比较（表 4）：两组治疗前 FEV1、FVC、FEV1/FVC 比较差异均无统计学意义（均 $P>0.05$）。两组治疗后 FEV1、FVC、FEV1/FVC 均较治疗前明显升高，且以观察组的升高程度较对照组更显著（均 $P<0.05$）。

表 4 不同治疗方法两组 AECOPD Ⅱ型呼衰患者治疗前后肺功能指标的比较（$\bar{x} \pm s$）

组别	时间	例数（例）	FEV1（L）	FVC（L）	FEV1/FVC（%）
对照组	治疗前	42	0.78±0.19	1.82±0.34	43.05±5.18
	治疗后	42	1.53±0.35[a]	2.57±0.43[a]	59.14±5.34[a]
观察组	治疗前	42	0.80±0.21	1.86±0.36	43.86±4.95
	治疗后	42	1.73±0.41[ab]	2.80±0.48[ab]	61.98±5.67[ab]

注：对照组给予无创机械通气，观察组行 HFNC；与本组治疗前比较，[a]$P<0.05$；与对照组比较，[b]$P<0.05$

2.4 两组发生并发症的发生情况和 30 d 内气管插管、病死率的比较（表 5）：观察组咽干、面部压伤、腹胀的发生率明显低于对照组（均 $P<0.05$）；两组患者气管插管率和病死率比较差异均无统计学意义（均 $P>0.05$）。

表 3 不同治疗方法两组 AECOPD Ⅱ型呼衰患者治疗前后 HR、RR 及动脉血气指标的比较（$\bar{x} \pm s$）

组别	时间	例数（例）	HR（次 /min）	RR（次 /min）	PaO_2（mmHg）	$PaCO_2$（mmHg）	SaO_2	pH 值
对照组	治疗前	42	116.55±6.68	30.40±3.67	55.33±6.43	70.17±8.37	0.87±0.09	7.24±0.08
	治疗后	42	93.57±4.77[a]	22.45±4.31[a]	76.21±8.23[a]	62.83±6.29[a]	0.92±0.04[a]	7.32±0.06[a]
观察组	治疗前	42	115.93±8.27	29.95±3.41	54.76±5.96	71.36±7.19	0.86±0.08	7.25±0.07
	治疗后	42	90.14±5.66[ab]	21.90±4.88[a]	80.70±7.81[ab]	60.02±5.93[ab]	0.94±0.04[a]	7.36±0.05[ab]

注：对照组给予无创机械通气，观察组行 HFNC；与本组治疗前比较，[a]$P<0.05$；与对照组同期比较，[b]$P<0.05$；1 mmHg≈0.133 kPa

表 5 不同治疗方法两组 AECOPD Ⅱ型呼衰患者并发症发生情况和 30 d 气管插管率、病死率的比较

组别	例数（例）	并发症发生率〔%(例)〕咽干	面部压伤	腹胀	气管插管率〔%(例)〕	病死率〔%(例)〕
对照组	42	19.0(8)	14.3(6)	26.2(11)	26.19(11)	7.14(3)
观察组	42	4.8(2)[a]	0.0(0)[a]	9.5(4)[a]	11.90(5)	4.76(2)

注：对照组给予无创机械通气，观察组行 HFNC；与对照组比较 [a]$P<0.05$

3 讨 论

AECOPD 呼出气流明显受限，通气障碍加重，导致呼衰，严重时可危及患者生命。部分患者需要气管插管行有创机械通气，但有创机械通气存在费用高、并发症多、脱机困难等问题，如何减少插管上机率是医务工作者一直思考的问题。无创机械通气临床已应用多年，对 AECOPD 合并呼衰患者的疗效也得到肯定，但在应用过程中受多种因素影响，如气道密封性、紧固性差，人机需保持同步呼吸，要求患者配合性较高，对呼吸道分泌物清除较困难，导致患者不能保证足够的上机时间，严重影响疗效。

呼吸道湿化治疗仪能为患者输送经过加温、湿化的高流量氧气，可用于有完全自主呼吸功能和建立人工气道的患者[10]。该治疗仪使用有弧度的鼻导管可减轻高速气流引起的额窦及呼吸道不适，不使用面罩，可减轻患者在治疗中出现的焦虑感，而且能提供大于患者通气量的气体流量，使患者吸入气体不被额外的空气稀释，保证了吸入氧气浓度的恒定[11]，加温、湿化功能可有效预防患者呼吸道水分丢失，使呼吸道分泌物更容易排出，降低患者气道敏感度及吸气阻力，进而改善肺顺应性，提高气体传导效率，其产生的 PEEP 可减少患者呼吸肌作功[12]，延长呼吸时间，从而提高肺泡通气量，达到增加肺通气效率的目的，具有精准调节 FiO_2 和不受患者自主呼吸影响的优点，可有效降低并发症发生率[13]。

本研究结果显示，经过 7 d 的治疗疗效显著，观察组与对照组 HR、PaO_2、$PaCO_2$、SaO_2、pH 值、FEV1、FVC、FEV1/FVC 比较差异均有统计学意义，且观察组咽干、面部压伤、腹胀的发生率均明显低于对照组，两组 RR、30 d 气管插管、病死率比较差异均无统计学意义。李林等[14]回顾性分析 60 例 AECOPD 合并Ⅱ型呼衰患者的临床资料，发现治疗后两组患者 pH 值差异无统计学意义，但 HR、RR、PaO_2、SaO_2 均较治疗前明显升高，$PaCO_2$ 较治疗前明显下降，且观察组的变化较对照组更显著；除 pH 值、RR 对比与本研究结论不一致外，其余结果均与本研究结论一致，李霜[15]在比较 120 例 AECOPD 患者的临床资料时发现，对于 pH 值、PaO_2、$PaCO_2$ 的改善，HFNC 优于无创机械通气，并且这种优势会随着时间的延长更加明显。说明 HFNC 治疗 AECOPD 合并Ⅱ型呼衰患者，无论疗效及舒适性、并发症发生率等方面均优于无创机械通气；同时 HFNC 在治疗创伤相关肺损伤中也在氧合指数的改善、机械通气时间、ICU 住院时间、舒适度评分等方面均优于无创机械通气[16]。

利益冲突 所有作者均声明不存在利益冲突

参考文献

[1] Wang C, Xu JY, Yang L, et al. Prevalence and risk factors of chronic obstructive pulmonary disease in China (the China Pulmonary Health [CPH] study): a national cross-sectional study [J]. Lancet, 2018, 391 (10131): 1706–1717. DOI: 10.1016/S0140-6736(18)30841-9.

[2] 文富强，陈磊. 中国慢性阻塞性肺疾病诊疗的现存问题与思考 [J]. 中华医学杂志, 2020, 100 (2): 81–84. DOI: 10.3760/cma.j.issn.0376-2491.2020.02.001.

[3] 范慧，索涛，赵乾秀，等. 经鼻高流量氧疗与无创正压通气治疗慢性阻塞性肺疾病急性Ⅱ型呼吸衰竭的比较 [J]. 武汉大学学报（医学版）, 2020, 41 (2): 291–295. DOI: 10.14188/j.1671-8852.2019.0482.

[4] 王超平，黄秀丽，罗晓斌，等. 经鼻高流量氧疗和无创机械通气在治疗新型冠状病毒肺炎所致急性呼吸窘迫综合征的比较研究 [J]. 中国呼吸与危重症监护杂志, 2022, 21 (1): 20–24. DOI: 10.7507/1671-6205.202006015.

[5] 张昌红，张作清，刘雪梅. 经鼻高流量吸氧治疗慢性阻塞性肺疾病急性加重期患者的临床效果分析 [J]. 现代诊断与治疗, 2021, 32 (9): 1429–1430.

[6] 吴国庆. 两种氧疗方式对 AECOPD 患者舒适度的比较研究 [J]. 江苏科技信息, 2021, 38 (29): 58–60. DOI: 10.3969/j.issn.1004-7530.2021.29.017.

[7] 张梅，王美堂. 经鼻导管高流量吸氧在慢性阻塞性肺疾病加重期的应用进展 [J]. 临床急诊杂志, 2020, 21 (7): 599–602. DOI: 10.13201/j.issn.1009-5918.2020.07.019.

[8] 中华医学会，中华医学会杂志社，中华医学会全科医学分会，等. 慢性阻塞性肺疾病基层诊疗指南（实践版·2018）[J]. 中华全科医师杂志, 2018, 17 (11): 871–877. DOI: 10.3760/cma.j.issn.1671-7368.2018.11.003.

[9] 中华医学会呼吸病学分会慢性阻塞性肺疾病学组. 慢性阻塞性肺疾病诊治指南（2013 年修订版）[J/CD]. 中国医学前沿杂志（电子版）, 2014, 6 (2): 67–80.

[10] 杨圣强，张贵真，刘贞，等. 经鼻高流量氧疗对慢性阻塞性肺疾病急性加重患者膈肌功能的影响：一项前瞻性随机对照研究 [J]. 中华危重病急救医学, 2019, 31 (5): 551–555. DOI: 10.3760/cma.j.issn.2095-4352.2019.05.006.

[11] 董好先. 经鼻高流量氧疗在老年慢性阻塞性肺疾病中的应用 [J]. 中国社区医师, 2021, 37 (25): 9–10. DOI: 10.3969/j.issn.1007-614x.2021.25.004.

[12] 张鹏，李争，江海娇，等. ICU 机械通气患者拔管后应用经鼻高流量序贯氧疗的效果分析 [J]. 中华危重病急救医学, 2021, 33 (6): 692–696. DOI: 10.3760/cma.j.cn121430-20210116-00074.

[13] 陈学昂，李素云，王明航，等. 慢性阻塞性肺疾病急性加重期预后影响因素的研究进展 [J]. 中华中医药学刊, 2017, 35 (4): 799–802. DOI: 10.13193/j.issn.1673-7717.2017.04.007.

[14] 李林，刘璐，何芳. 经鼻高流量湿化氧疗治疗 AECOPD 合并Ⅱ型呼吸衰竭的疗效分析 [J/CD]. 中华肺部疾病杂志（电子版）, 2021, 14 (4): 422–426. DOI: 10.3877/cma.j.issn.1674-6902.2021.04.004.

[15] 李霜. 经鼻高流量湿化氧疗对 AECOPD 患者血气指标、肺功能的影响 [J]. 微量元素与健康研究, 2022, 39 (1): 13–16.

[16] 毛越，窦琳. 经鼻高流量氧疗与无创正压通气治疗创伤性相关性肺损伤的临床对比研究 [J]. 中国中西医结合急救杂志, 2021, 28 (6): 668–671. DOI: 10.3969/j.issn.1008-9691.2021.06.005.

（收稿日期：2023-04-20）
（责任编辑：邸美仙）
