# Supplementary figures and images for: High‐Flow Nasal Cannula in Hypercapnic Respiratory Failure: An Updated Systematic Review and Meta‐Analysis
Source: Clin Respir J. 2026 Jul 1;20(7):e70207. doi: 10.1111/crj.70207 (PMC13323174; doi:10.1111/crj.70207)

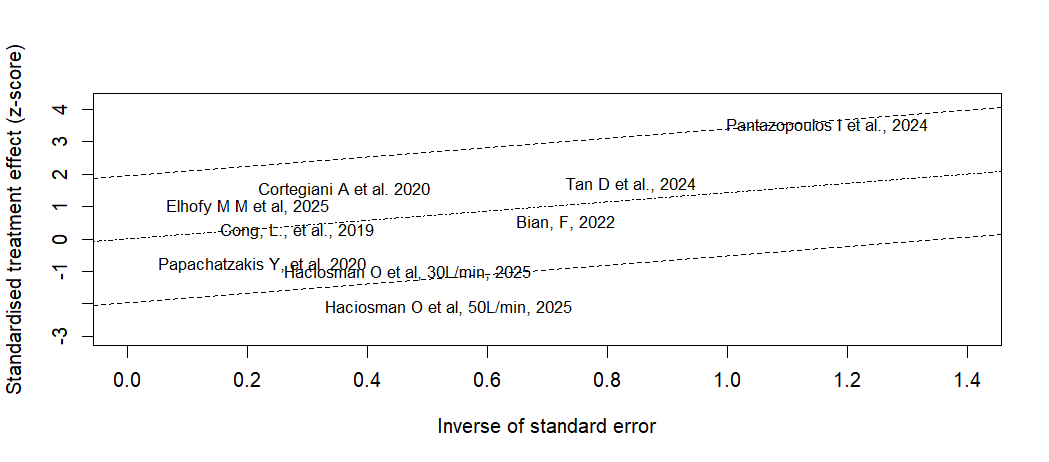

Supplement: Supplementary file 2 — Data S2: Supporting information. [file CRJ-20-e70207-s003.zip › Figure S/Figure S1 Galbraith radial plot for CO2.jpg]

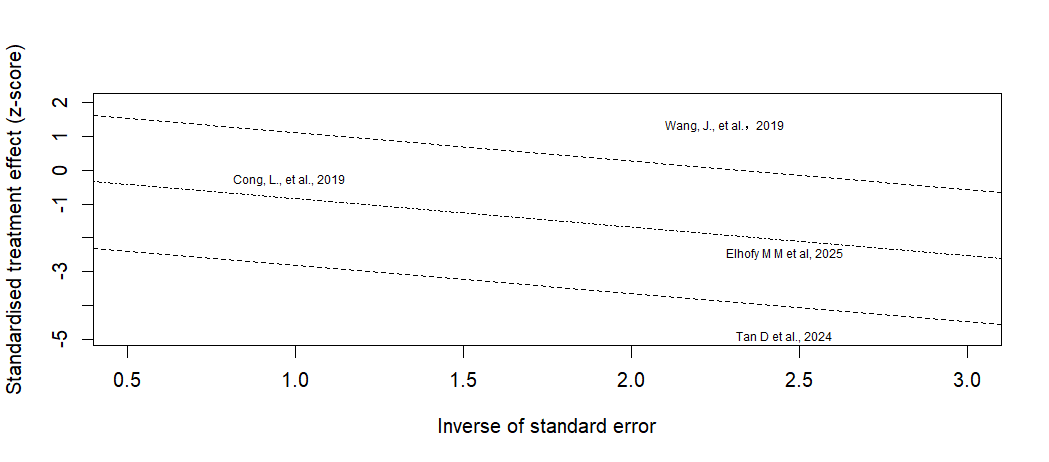

Supplement: Supplementary file 2 — Data S2: Supporting information. [file CRJ-20-e70207-s003.zip › Figure S/Figure S2 Galbraith radial plot for Stayo f ICU.png]

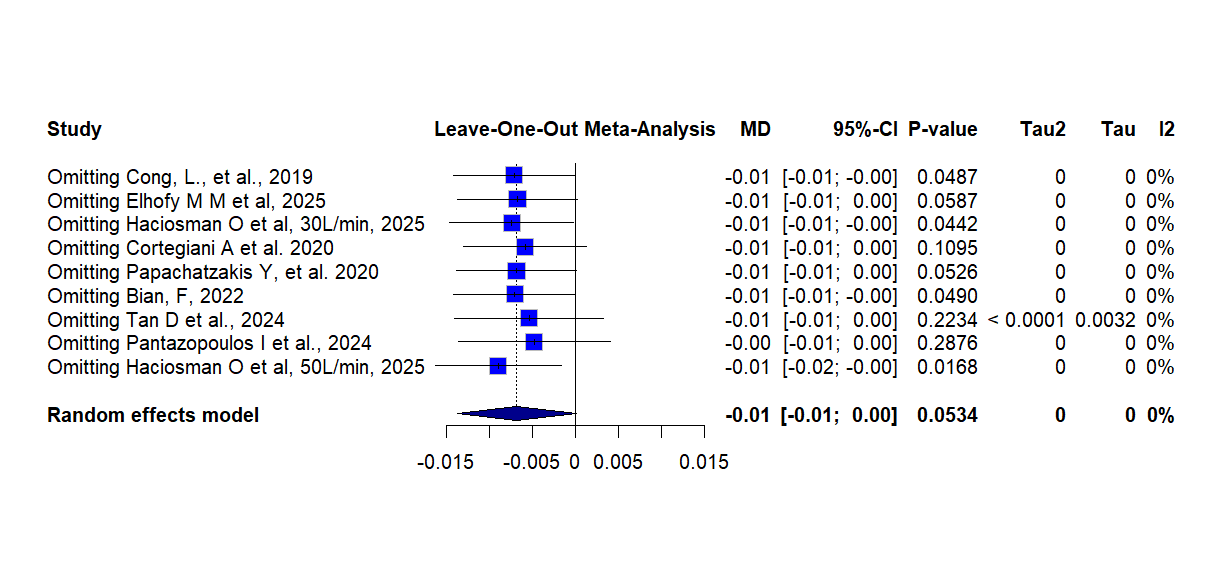

Supplement: Supplementary file 2 — Data S2: Supporting information. [file CRJ-20-e70207-s003.zip › Figure S/Figure S3 sensitivity_pH.jpg]

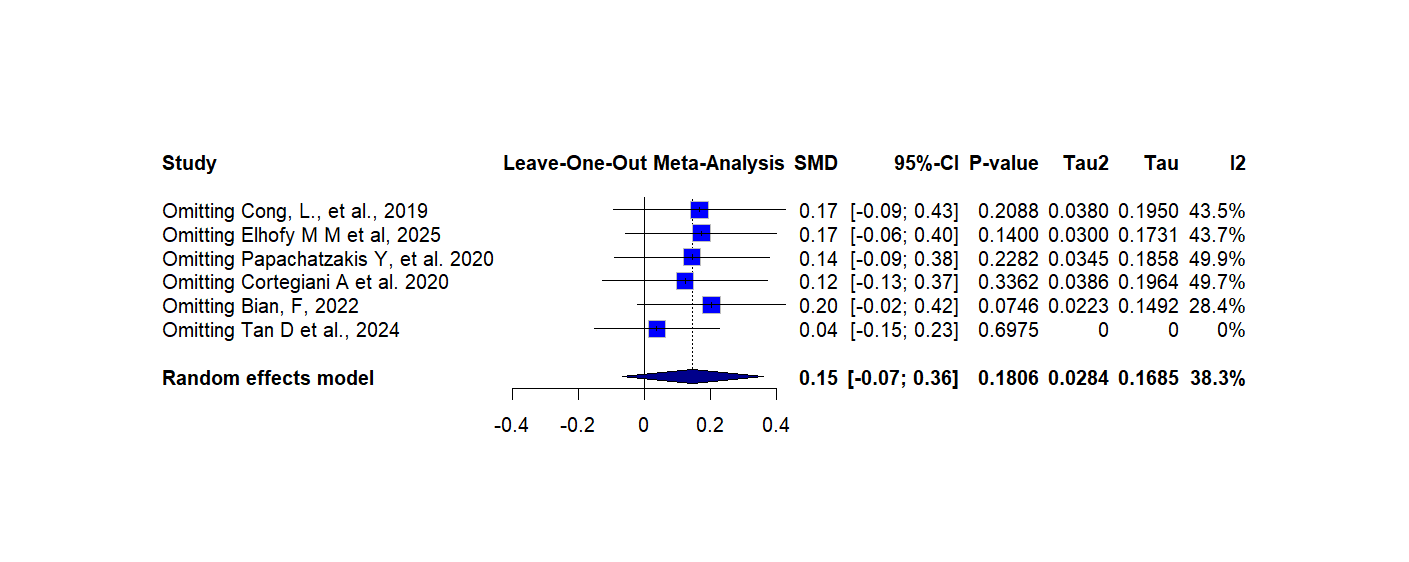

Supplement: Supplementary file 2 — Data S2: Supporting information. [file CRJ-20-e70207-s003.zip › Figure S/Figure S4 sensitivity_PaO2.jpg]

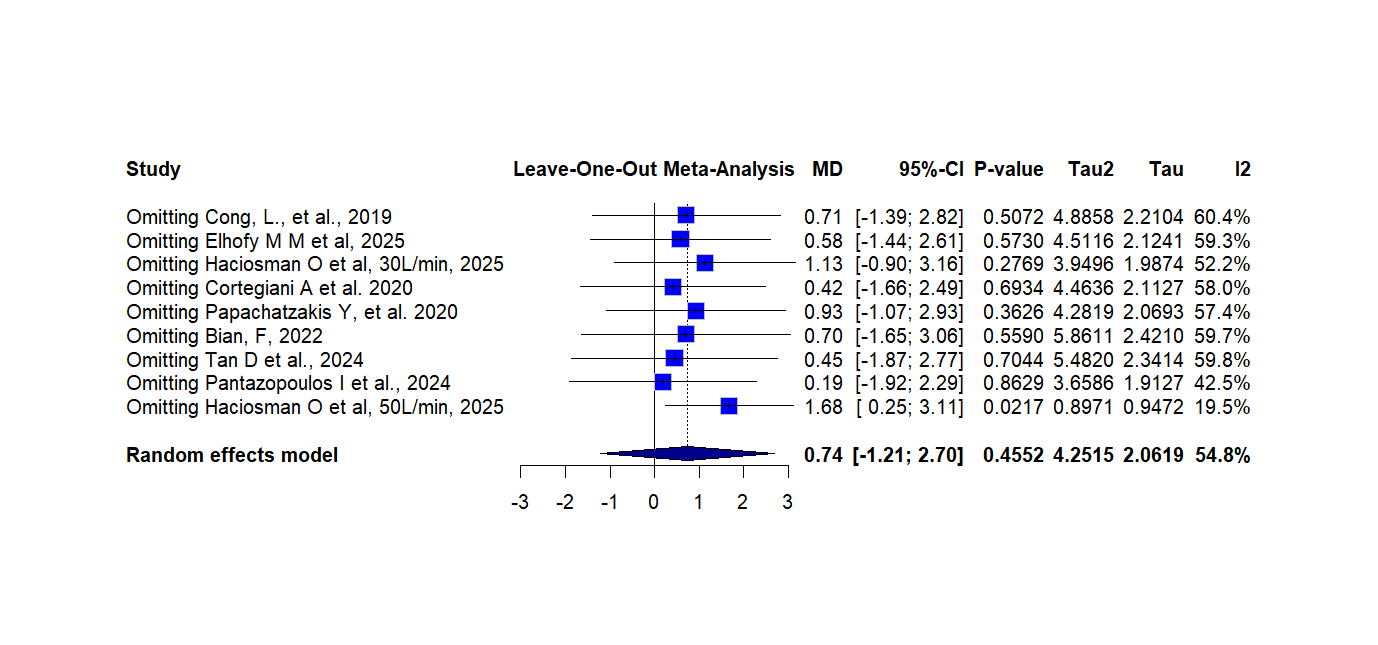

Supplement: Supplementary file 2 — Data S2: Supporting information. [file CRJ-20-e70207-s003.zip › Figure S/Figure S5 sensitivity_PaCO2.jpg]

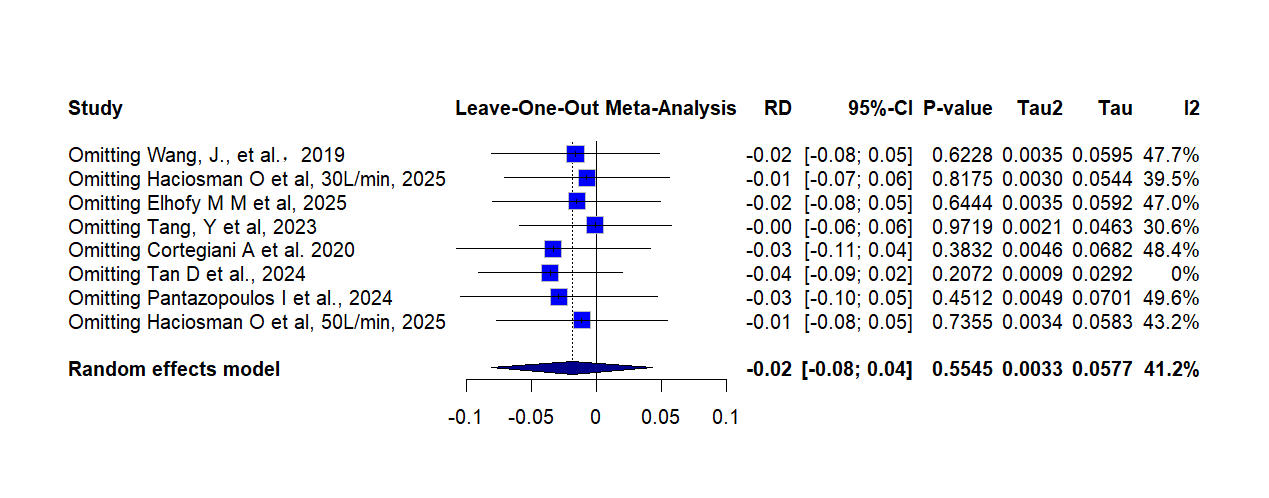

Supplement: Supplementary file 2 — Data S2: Supporting information. [file CRJ-20-e70207-s003.zip › Figure S/sensitivity_intubation_plot_zoom_png.width=1024&height=402.jpg]

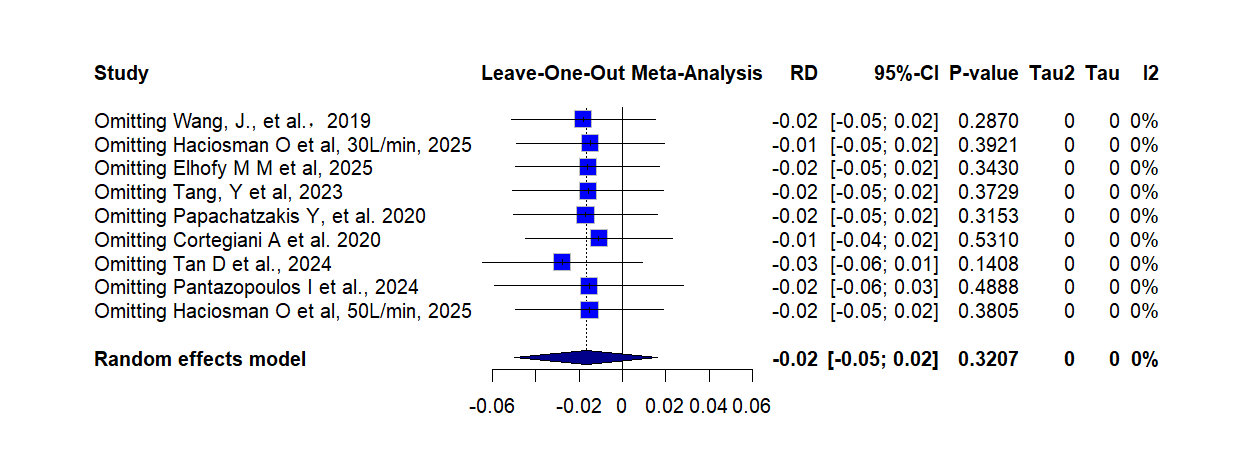

Supplement: Supplementary file 2 — Data S2: Supporting information. [file CRJ-20-e70207-s003.zip › Figure S/sensitivity_mortality_plot_zoom_png.width=1012&height=366.jpg]
